# Heterotrimeric G-proteins and cAMP regulate gene expression during growth on cellulose in *Neurospora crassa*

**DOI:** 10.1128/mbio.03720-25

**Published:** 2026-01-15

**Authors:** Logan Collier, Yagna Oza, Monique Quinn, Alexander J. Carrillo, May M. Campbell, Katherine A. Borkovich

**Affiliations:** 1 Department of Microbiology and Plant Pathology, University of California 207030, Riverside, California, USA; Instituto Carlos Chagas, Curitiba, Brazil

**Keywords:** G proteins, cellulase, transcriptomics, adenyl cyclases, cyclic AMP, filamentous fungi, *Neurospora*

## Abstract

Aspects of transcriptional regulation of plant cell wall-degrading enzyme (PCWDE) genes have been characterized in the filamentous fungus *Neurospora crassa*. However, the upstream signaling pathways that regulate PCWDE expression are not well understood. We have previously reported roles for heterotrimeric G-proteins and adenylyl cyclase in the degradation of cellulose to glucose in *N. crassa*. Here, we performed mRNA-seq to identify patterns of gene expression after transfer from glucose to cellulose medium in wild type, the Gα mutants Δ*gna-1* and Δ*gna-3,* and the adenylyl cyclase mutant Δ*cr-1*. In wild type, 3719 genes were regulated at least twofold during growth on cellulose vs glucose. Analysis of transcriptomics data for the strains after transfer from glucose to cellulose demonstrated that the Δ*cr-1* mutant had the most misregulated genes, with 2,232, followed by Δ*gna-3* with 1,182 and Δ*gna-1* with 648 genes. Metabolic genes were the most prevalent differentially expressed genes in the mutants. Expression of PCWDEs, including most of the cellulases, was downregulated in the three mutants, with Δ*cr-1* displaying the greatest deficiency. Furthermore, several transcription factors essential for cellulase expression were misregulated in the mutants. The primary factors *clr-1* and *clr-2* were downregulated in Δ*gna-3* and Δ*cr-1* strains, and *clr-2* was reduced in Δ*gna-1* mutants. Overexpression of *clr-2* restored cellulase activity and increased the expression of two major cellulase genes in all three mutants. Taken together, our results demonstrate that heterotrimeric G-proteins and cAMP signaling strongly impact transcriptional control of cellulase activity, culminating in the expression of the transcription factor *clr-2* in *N. crassa*.

**IMPORTANCE:**

Filamentous fungi are important organisms for degradation of plant biomass. Both nonpathogens and plant pathogens secrete plant cell wall degrading enzymes to release simple sugars from the plant cell wall to use as carbon sources for growth. Much is known about the transcription factors that control production of plant cell wall-degrading enzymes by fungi. However, mechanistic details for how different lignocellulosic compounds are sensed by these organisms and the resultant cellular responses that operate upstream of cellulase-regulating transcription factors are lacking. Our research helps bridge this gap by identifying the role of G-protein subunits and cAMP in the regulation of gene expression during growth on cellulose. Understanding the environmental sensing and signal transduction pathways that regulate cellulase gene expression will have applications to agricultural losses due to plant pathogens, carbon recycling in the environment, and production of biofuels.

## INTRODUCTION

The polysaccharide cellulose is an integral part of the plant cell wall structure (1). Cellulose is composed of glucose monomers joined in β-1,4-linkages, with weak hydrogen bonds tethering neighboring chains. Saprophytic and plant pathogenic fungi degrade cellulose through the secretion of an enzyme cocktail containing several plant cell wall-degrading enzymes (PCWDEs), including multiple enzyme families specifically for cellulose degradation (2). Endoglucanases, cellobiohydrolases, β-glucosidases, cellobiose dehydrogenases, and polysaccharide monooxygenases comprise major cellulase families in filamentous fungi such as *Neurospora crassa*. Endoglucanases cleave cellulose chains using a hydrolytic reaction (3) to create reducing ends, while polysaccharide monooxygenases cooperate with cellobiose dehydrogenases to achieve the same result through an oxidative process (4–6). Cellobiohydrolases release cellobiose (a glucose dimer) molecules from the open ends (7, 8), and β-glucosidases hydrolyze cellobiose to form two glucose monomers (9). Cellobiose and glucose can then be transported into the cell to be used as carbon sources (10).

Multiple proteins that regulate plant cell wall degradation have been identified in the filamentous fungus *N. crassa* (11–15). The C2H2 zinc finger transcription factor CRE-1 regulates carbon catabolite repression (CCR). CCR suppresses the expression of genes required for utilizing alternative carbon sources (e.g., cellulose) in the presence of a preferred carbon source such as glucose (16, 17). In *N. crassa,* CRE-1 represses the expression of four different target gene groups that regulate cellulolytic gene expression: the transcription factor *clr-1* (see below), two cellodextrin transporter genes, a few PCWDEs, and numerous Major Facilitator Superfamily (MFS) transporter genes (17). The NDT80 class transcription factor VIB-1 is crucial for the regulation of cellulase production under inductive conditions by repressing CRE-1-mediated CCR and glucose sensing/metabolism by the fungal-specific C6 finger domain transcription factor COL-26 (17, 18). In contrast, COL-26 alleviates CCR regulation by inhibiting CRE-1 activity in the presence of cellulose (18).

In *N. crassa*, cellulose breakdown is regulated by a group of five proteins identified as cellulose degradation regulators (CLRs) (12–15, 19, 20). CLR-1, CLR-2, CLR-4, and CLR-5 are fungal-specific C6 finger domain transcription factors (File S2) (21), while CLR-3 is a DUF1479 domain-containing protein without obvious DNA-binding regions (13). CLR-1 is activated by cellobiose through an unknown mechanism (20) and regulates the expression of *clr-2*, β-glucosidases, sugar transporters, and other genes (17). CLR-2 drives the expression of numerous cellulase genes (12, 17). CLR-4 is required for transcription of *clr-1* and *clr-2*, as well as adenylyl cyclase (14). CLR-3 was identified as a negative regulator of *clr-1* expression (13). The observation that the expression of *clr-3* is increased on cellulose and its promoter is bound by CLR-1 and CLR-2 suggests self-negative regulation by CLR-1 (13). CLR-5 plays a role in cellulose degradation through the regulation of amino acid metabolism in *N. crassa* (15).

While many transcription factors involved in cellulose degradation have been identified, less is known about upstream regulators. We have published two studies that demonstrate a role for heterotrimeric G-protein signaling in cellulose degradation in *N. crassa* (22, 23). First, a global analysis of all G-protein-coupled receptors (GPCRs) demonstrated that three GPCR mutants possessed growth defects on cellulose and that multiple GPCR genes were more highly expressed on Avicel (crystalline cellulose) than on sucrose (22). Second, we showed that five of the six G-protein subunits (Gα genes *gna-1* and *gna-3*, Gβ genes *gnb-1* and *cpc-2*, and the Gγ gene *gng-1*) and the adenylyl cyclase *cr-1* are required for normal cellulase activity in *N. crassa* (23). Using quantitative real-time PCR (qRT-PCR), we also demonstrated that the transcript levels for certain key cellulase enzymes were significantly reduced in the Δ*gna-1*, Δ*gna-3*, Δ*gnb-1,* and Δ*cr-1* mutants (23). Furthermore, cAMP supplementation increased the cellulase activity and transcript levels for the five cellulase genes in the same four mutants.

In this study, we use mRNA-seq to explore the effects of G-protein signaling on global patterns of gene expression in wild-type, Δ*gna-1*, Δ*gna-3*, and Δ*cr-1* strains after transfer from glucose to cellulose. We identified misregulated genes in the three mutants on cellulose, including reduced levels of PCWDE mRNAs. We observed downregulation of transcription factors that regulate cellulase expression, with *clr-2* mRNA levels reduced in all mutants. We overexpressed *clr-2* in wild type and the mutants and observed increased cellulase activity and expression of two cellulase genes. Our results are consistent with positive regulation of *clr-2* expression and downstream cellulase production by G-protein signaling and cAMP levels in *N. crassa*.

## RESULTS

### Cellulase activity of wild-type, Δ*gna-1,* Δ*gna-3,* and Δ*cr-1* strains after a 2-day transfer experiment

**Fig 1:**
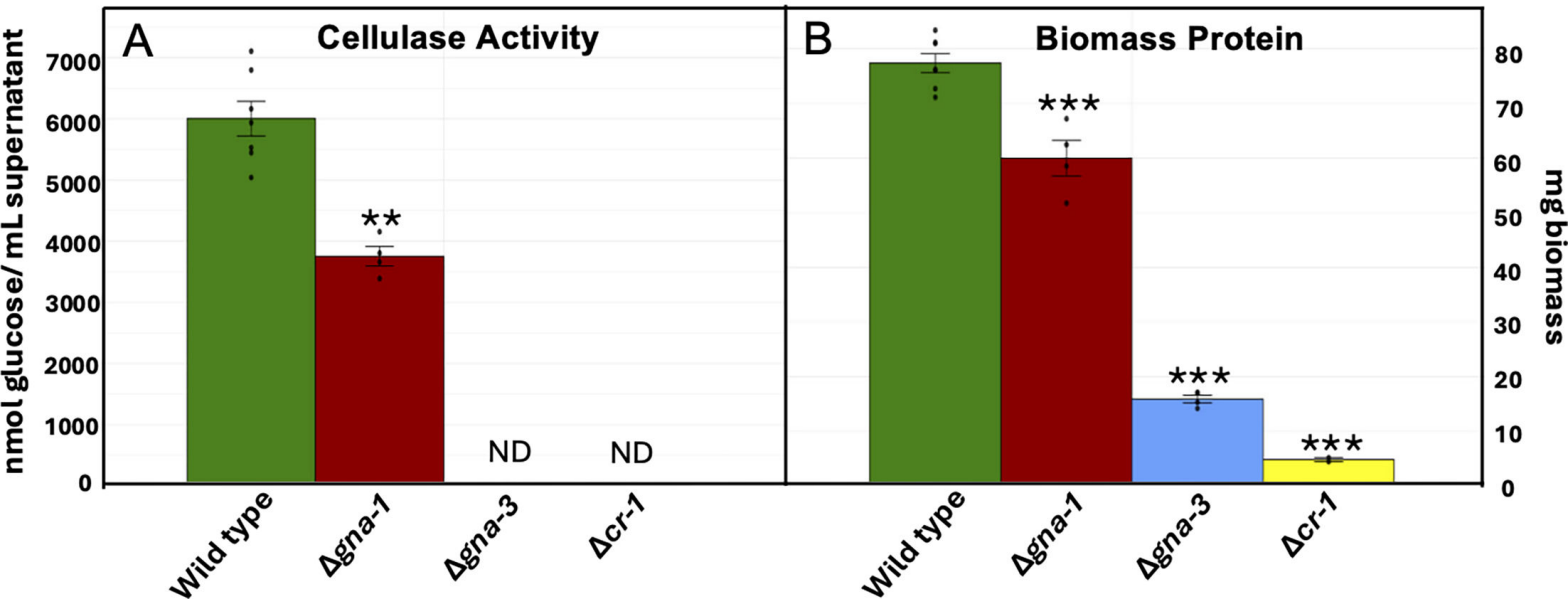
Cellulase activity and biomass protein after a 2-day transfer experiment. Strains were cultured in medium with glucose as a carbon source for 16 h, and the mycelium was collected, washed, and then transferred to Avicel medium for 48 h. A sample of each culture supernatant was withdrawn and passed through a 0.45-µm filter. A minimum of three biological replicates were used for all assays, and the error is expressed as the standard error. Individual data points are indicated as black dots. Statistical significance relative to wild type was determined using a two-tailed Student’s *t*-test, and strains with protein levels or cellulase activity significantly different from that of the wild type are indicated as *P < 0.05, **P < 0.01, and ***P < 0.001. (**A**) Cellulase activity. The ability of cell-free supernatants to degrade Avicel to glucose (cellulase activity) was measured as described in Materials and Methods. Units are expressed as nmol glucose/mL culture supernatant. ND = no detectable activity. (**B**) Biomass protein. Total protein was extracted from cell pads of each culture, as described in Materials and Methods. Samples were assayed using the BCA protein assay. The total protein (mg) was calculated using the total volume of the extract.

We previously measured Avicelase (cellulase) activity in the secretomes from strains grown in glucose medium for 16 h and then transferred to Avicel medium for 3 days (23). However, since wild type exhausts the Avicel in the medium by 3 days and may be experiencing carbon starvation, we transitioned to 2 days of growth in Avicel, under which conditions residual Avicel is present in wild-type cultures. We have also recently noted that clumping of cultures (fungal ball formation) leads to low or undetectable cellulase activity, perhaps due to reduced carbon utilization (24). Therefore, any clumped cultures were discarded and not utilized for our analysis. Using these new parameters, we measured cellulase activity in wild-type, Δ*gna-1,* Δ*gna-3*, and Δ*cr-1* strains (Table S1; Fig. 1A). As previously shown for 3-day transfer experiments, the wild-type strain exhibits robust activity, while the Δ*gna-3* and Δ*cr-1* strains have no detectable activity (23). However, the Δ*gna-1* mutant exhibits cellulase activity that is 62.5% of wild type under these new conditions, suggesting that clumping likely played a role in the lack of activity noted previously (23). The total biomass protein levels in the Δ*gna-1,* Δ*gna-3*, and Δ*cr-1* mutants are significantly reduced compared to those in the wild type (Fig. 1B), with Δ*gna-3* and Δ*cr-1* strains displaying a more severe growth defect relative to the Δ*gna-1* mutant.

### Identification of differentially expressed genes in wild type during growth on cellulose vs. glucose

We previously analyzed the mRNA transcript levels of five major cellulase genes in wild-type, Δ*gna-1,* Δ*gna-3*, and Δ*cr-1* strains through qRT-PCR experiments and observed reductions in all three mutants (23). To decipher the full extent of transcriptional regulation by *gna-1, gna-3*, and *cr-1* in the growth of *N. crassa* on cellulose, we performed mRNA sequencing. We now grew cultures for 12 h in 2% glucose, washed twice in no carbon medium, and then transferred to either 2% glucose or 2% cellulose (Avicel) for 4 h to capture early changes after the transition to cellulose. It should be noted that we cannot distinguish defects in derepression due to the absence of glucose vs defects in induction by cellulose in our strains using our data. RNA was isolated from the strains and used to prepare libraries for RNA-seq. Principal component analysis (PCA) demonstrated that the three biological replicates for each strain grown on the same medium clustered together and that the replicates for the four strains were well-separated (Fig. S1).

**Fig 2:**
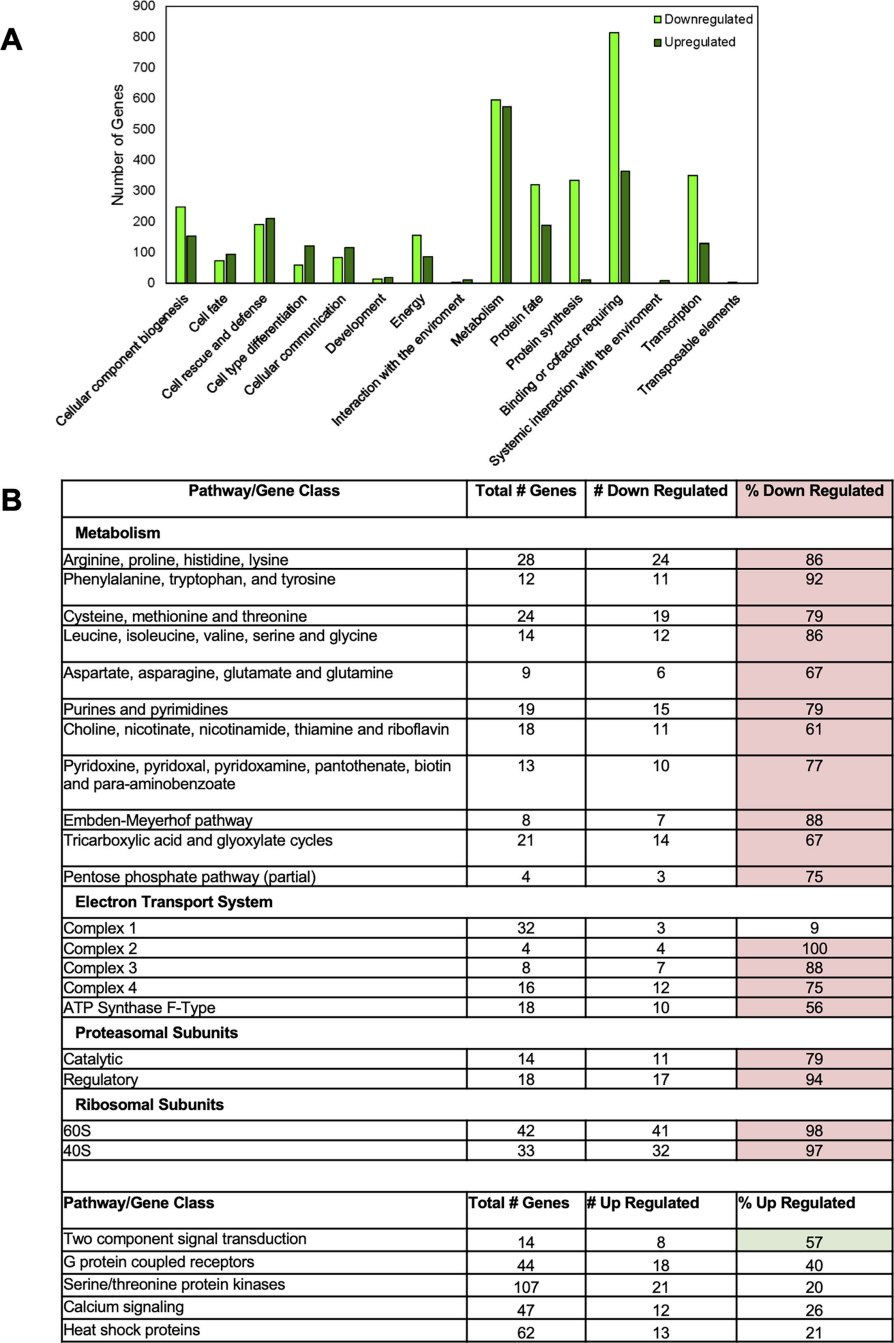
Differentially regulated genes in wild type during growth on cellulose vs glucose medium. Wild type was cultured for 12 h in glucose followed by washing and transfer to cellulose (Avicel) or glucose for 4 h. RNA was isolated and used to prepare libraries for RNA-seq, as described in Materials and Methods. Genes with ≥10 transcripts per million (TPMs) and those that differed significantly from glucose cultures with a fold change of ≥2.0 were included. (**A**) Functional categories for differentially expressed genes in wild type cultured on cellulose vs glucose. Functional catalog analysis (level 1 categories) was performed as described in Materials and Methods. Data are in File S1. (**B**) Examples of differentially down- and upregulated pathways during growth on cellulose vs glucose. Instances where the number of misregulated genes is greater than or equal to 50% are indicated in red (downregulated relative to wild type) and green (upregulated relative to wild type).

We began by comparing wild-type cultures grown with glucose as the carbon source to those cultured with glucose and then transferred to cellulose for 4 h (File S1). Using DESeq2, we observed that 3,719 genes were differentially expressed at twofold or greater levels on cellulose vs glucose, with 1,801 downregulated and 1,918 upregulated, on cellulose. We used FungiFun2 (25) to determine functional category associations (Level 1) for those genes that were differentially expressed in wild type in cellulose relative to glucose (Fig. 2A). For the genes with increased expression on cellulose (upregulated), Metabolism was the largest category, followed by Binding or Cofactor Requiring, Cell Rescue and Defense, and Protein Fate (Fig. 2A). For downregulated genes, Binding or Cofactor Requiring was the largest category, followed by Metabolism and Transcription (Fig. 2A).

The downregulated genes in wild type after transfer to cellulose encompassed numerous pathways for primary metabolism, including most amino acid biosynthetic pathways, fatty acid metabolic genes, components in complexes 2–4 of the electron transport system, and the mitochondrial F1 ATP synthase (Fig. 2B; File S2). There was extensive downregulation of most ribosomal protein subunits and several protein factors that regulate translation (Fig. 2B; File S2). Furthermore, expression of numerous protein chaperones (including most subunits of the chaperonin containing Tailless complex [CCT]) (26) and proteasomal subunits was decreased after transfer to cellulose. Of interest, a major protein client of CCT is tubulin, which was downregulated 3.5-fold in wild type during growth on cellulose (File S2). Taken together, the downregulated genes on cellulose suggest a decrease in primary metabolism and reduced protein translation, which may lessen the need for quality control of protein folding.

Numerous genes implicated in environmental sensing were expressed more highly in wild type cultured on cellulose vs glucose (File S2), including members of two-component regulatory systems (27), GPCRs (22), serine-threonine protein kinases (28), and several proteins required for hyphal anastomosis/fusion (*ham-6*, *ham-7*, *ham-8*, *ham-9*, *ham-13*, *ham-14*, and *mik-1*) (29–32) or self/non-self recognition (*doc-2*) (33) in *N. crassa* (Fig. 2B; File S1). Notably, a regulator of G-protein signaling gene (NCU05435/*rgs-2*) that is required for normal cellulase activity (34) is upregulated more than 15-fold on cellulose (File S1). Expression of both cAMP-phosphodiesterase (cAMP-PDE) genes (NCU00478/*acon-2* and NCU00237/*pde-1*) were upregulated on cellulose (File S1). These findings suggest that cellular signaling and cell-cell interactions are increased during growth on cellulose. In contrast, among heterotrimeric G-protein subunits, only the Gβ *cpc-2* was differentially expressed in wild type (7.6-fold downregulated on cellulose; File S1). Adenylyl cyclase (*cr-1*) and genes encoding the regulatory and catalytic subunits of PKA-/cAMP-dependent protein kinase (*mcb*, *pkac-1,* and *pkac-2*) were not differentially regulated (File S1). These observations reinforce the notion that pre-existing heterotrimeric G-protein subunits, adenylyl cyclase, and PKA may potentially participate in sensing of cellulose or other cellodextrins after the transfer from glucose to cellulose medium.

LAE-1/NCU00646, VE-1/NCU01731, and VE-2/NCU02775 are components of the velvet complex in *N. crassa*, which regulates asexual and sexual development and secondary metabolism (35, 36). Levels of *ve-1* and the three LaeA domain methyltransferase genes *lae-1*, NCU01148, and NCU05501, were elevated in wild type after the transfer to cellulose (File S1). Functions for *N. crassa lae-1* in regulating cellulase activity or expression have not been described (35), but LaeA orthologs have been implicated in temporal regulation of cellulase expression in several fungi, including *Myceliophthora thermophila* (15) and *Trichoderma reesei* (37). Therefore, increased expression of these genes may reflect gross changes in chromatin remodeling in wild type after the transition from glucose to the cellulose medium.

We noted upregulation of three of the six *N. crassa* photoreceptor genes (27, 38) in wild type after transfer to cellulose, including the green light sensing GPCR *nop-1* and the blue light-responsive proteins *wc-1* and *vvd* (File S2). VVD is homologous to ENVOY, which regulates cellulase gene expression in *Trichoderma* species (39). In *N. crassa*, VVD interacts with the white-collar complex (WCC, consisting of WC-1 and WC-2) and an RNA helicase to regulate the circadian clock (40). It has been demonstrated that mutants lacking *vvd*, *wc-1,* or *wc-2* have elevated levels of endoglucanase activity after 40 h of growth in Avicel in constant light (41). Inspection of the available microarray data (1.5-fold cutoff) revealed elevated expression of one endoglucanase gene in the Δ*vvd* mutant, but somewhat paradoxically, lower expression of three or two endoglucanase genes in the Δ*wc-1* and Δ*wc-2* mutants, respectively (41). The authors ascribed these results to the negative regulatory role of VVD in photoadaptation and the complex regulation of the WCC by light.

### The Δ*cr-1* adenylyl cyclase mutant possesses the most differentially regulated genes during growth on cellulose

**Fig 3:**
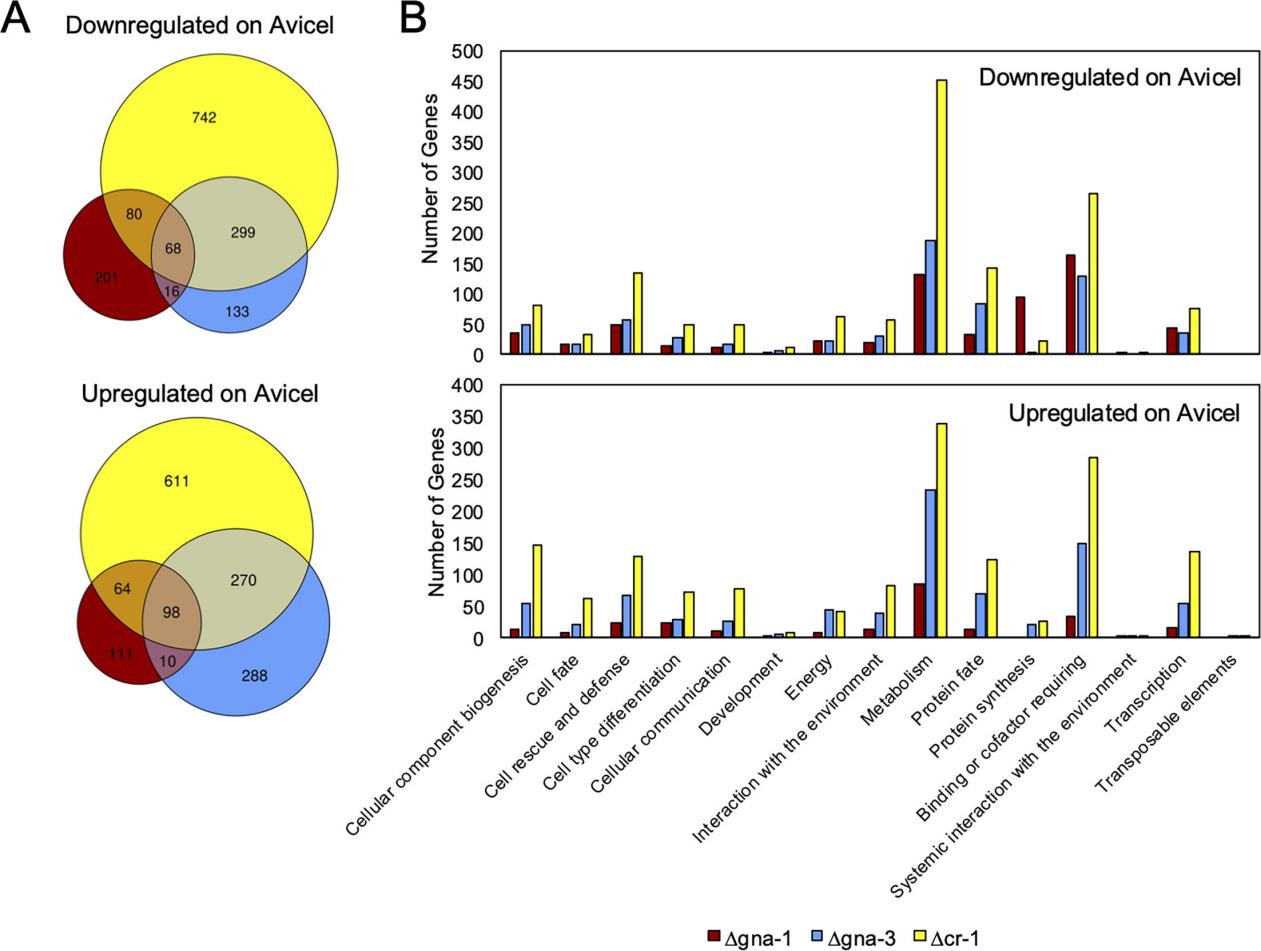
Numbers and functional categories for differentially expressed genes in the three mutants relative to wild type after transfer to cellulose. (**A**) Differentially expressed genes. The numbers in the Venn diagrams represent the number of shared differentially expressed genes that are downregulated (top) or upregulated (bottom) in one, both, or all three mutants as compared to wild type after transfer to cellulose. The red, blue, and yellow circles represent all differentially regulated genes in the Δ*gna-1*, Δ*gna-3,* and Δ*cr-1* mutants, respectively. Only genes that were significantly different than in wild type (with *P* < 0.05), with a fold change of 2 or higher were included. (**B**) Up- and downregulated functional categories. Functional catalog analysis was performed comparing wild type and the Δ*gna-1*, Δ*gna-3*, and Δ*cr-1* mutants for the level 1 categories. Data are in File S1.

We next compared the transcriptomes of wild type and three mutants after transfer to cellulose. The Δ*cr-1* mutant possessed the greatest number of down (1,189) and upregulated (1,043) genes (2,232 total) vs wild type. It should be noted that this strain has the most severe growth defect, which may indirectly influence the expression of some genes. Δ*gna-3* had 516 downregulated and 666 upregulated genes (1,182 total), followed by Δ*gna-1,* with 365 downregulated and 283 upregulated genes (648 total) (Fig. 3A). More than half of the downregulated genes in Δ*gna-1* and Δ*gna-3* mutants were shared with the Δ*cr-1* strain. This trend was also observed for upregulated genes in the Δ*gna-3* mutant, where 367 genes (71%) were also upregulated in Δ*cr-1*. Greater than 50% of the up- and downregulated genes were uniquely misregulated in the Δ*cr-1* mutant. This was also true for downregulated genes in the Δ*gna-1* mutant. Finally, only 68 downregulated and 98 upregulated genes were shared among the three mutants (Fig. 3A).

 We identified the functional categories that weremisregulated in the mutants compared to wild type during growth on cellulose. We analyzed the major (level 1) categories (Fig. 3B) and found that “Metabolism” and “Binding and Cofactor Requiring” were the largest in all mutants for up and downregulated genes (Fig. 3B). In addition, some unique differentially regulated categories were observed for each mutant. In the Δ*gna-1* mutant, “Protein Synthesis” was a major downregulated category (Fig. 3B), while “Protein Fate” was a prominent downregulated category in the Δ*gna-3* mutant. For the Δ*cr-1* strain, “Protein Fate” and “Cell Rescue and Defense” were significant categories for up and downregulated genes, while “Transcription” and “Cellular Component Biogenesis” were among upregulated gene categories (Fig. 3B).

### Expression of PCWDE genes, including key cellulase classes, is altered in the three mutants

**Fig 4:**
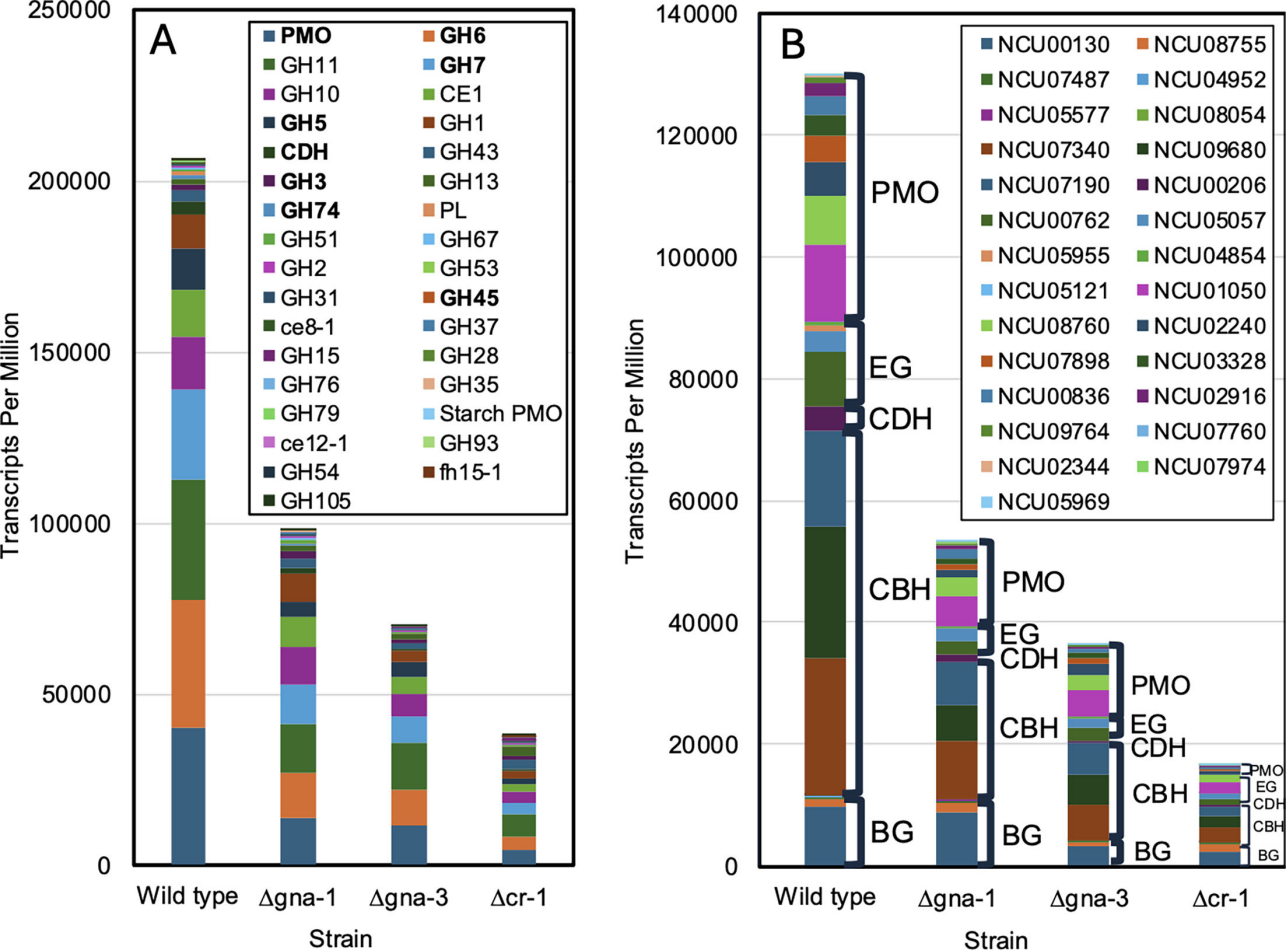
Expression of PCWDE and cellulase genes in the strains after transfer from glucose to cellulose. Average TPMs in the three biological replicates for aggregated PCWDE genes (**A**) or for individual cellulase genes (**B**) are shown, with the largest value for each PCWDE or cellulase class at the bottom of the bar for wild type in the figure. (**A**) Expression of PCWDEs. Relative expression of PCWDE genes in the four strains after growth on cellulose is shown. The cellulase gene families are indicated in bold font in the legend on the graph. Genes and classes were obtained from reference 17. (**B**) Expression of annotated cellulase genes. Relative expression of major classes of annotated cellulases in the four strains after growth on cellulose is shown. Gene numbers are indicated in the legend. BG, β-glucosidases; CBH, cellobiohydrolases; CDH, cellobiose dehydrogenases; EG, endoglucanases; PMO, polysaccharide monooxygenases.

Consistent with earlier studies (17, 19, 42), numerous PCWDEs were upregulated in wild type during growth on cellulose vs glucose (File S1). We used a curated list of 113 PCWDEs (17) to compare the total average TPMs of these genes in wild type and the three mutants after transfer to cellulose (Fig. 4A; File S2). The group of genes includes enzymes required for degradation of cellulose, hemicellulose, pectin, starch, and other sugars. All three mutants had greatly reduced TPMs relative to wild type, with Δ*cr-1* being most affected. The total TPMs in Δ*gna-1* mutants were 47.5% of wild type, while those in Δ*gna-3* and Δ*cr-1* strains were 33.9% and 18.4% of wild type, respectively (File S2). In Δ*cr-1*, there were several lower expressed enzyme classes that were only modestly reduced or even increased (File S2).

We focused on the genes implicated in cellulose degradation in more detail (Fig. 4B). Like what was observed for PCWDEs, total TPMs followed the trend Δ*gna-1* > Δ*gna-3* > Δ*cr-1*, with 40.9%, 28.0%, and 12.7% of wild-type levels, respectively. The trend in downregulated genes is consistent with transcriptional control of cellulase gene expression since Δ*cr-1* and Δ*gna-3* mutants do not have detectable cellulase activity (23) (Fig. 1). The greatest percent decrease across all three mutants was observed for cellobiose dehydrogenase (6.4%–30.5% of wild type). Slightly less reduction was observed for cellobiohydrolases, endoglucanases, and polysaccharide monooxygenases (Fig. 4B; File S2).

The trend in total TPMs reduced relative to the wild type was retained for the β-glucosidases in the Δ*gna-3* and Δ*cr-1* mutants, with levels only 36.5% and 33.4% of wild type. However, total TPMs for β-glucosidases were only modestly affected in Δ*gna-1* mutants (94.7% of wild type), and TPMs for four genes were elevated relative to the wild type (Fig. 4B; File S2). The latter observation may explain why significant cellulase activity is observed in Δ*gna-1* strains, despite the 30%–40% reduction in transcript levels for the other major cellulase gene classes in this mutant (Fig. 4B; File S2).

### Examples of misregulation of non-PCWDE gene classes in the three mutants

We noted differences in the expression of many other genes in the mutants during growth on cellulose. For example, analysis of predicted MFS small-molecule transporters (File S2) revealed that out of the three cellobiose transporter genes (NCU00801/*cdt-1*, NCU08114/*cdt-2,* and NCU05853/*cbt-1*) (20), *cdt-1* and *cbt-1* were downregulated in the Δ*cr-1* mutant, while only *cbt-1* was downregulated in the Δ*gna-3* mutant. Evidence suggests that CDT-1 and CDT-2 can function as both cellobiose sensors/transceptors and transporters (20), but similar information is not available for CBT-1. Downregulation of *cdt-1* and/or *cbt-1* could lead to reduced sensing and/or transport of the signaling molecule cellobiose (17, 20) into the Δ*cr-1* and Δ*gna-3* mutants.

We observed differential expression of numerous GPCRs and photoreceptors in the three mutants during growth on cellulose (Table S2). It is noteworthy that nearly half of the misregulated GPCRs in the three mutants belong to the Pth11-like class, as we previously demonstrated that three members of this group have growth phenotypes on Avicel (22) (Table S2). Four photoreceptor genes were misregulated in at least one mutant during growth on cellulose (Table S2). Since light is known to increase cellulase gene expression in *T. reesei* (43, 44) and *N. crassa* mutants lacking the *wc-1*, *wc-2,* or *vvd* genes have reduced expression of cellulases in light-grown cultures (41), upregulation of photoreceptor gene expression may be a compensatory mechanism in the mutants in our study.

Levels of both genes encoding the catalytic subunit of cAMP-dependent protein kinase (NCU06240/*pkac-1* and NCU00682/*pkac-2*) were not differentially regulated in wild type on cellulose vs glucose or in the mutants vs wild type during growth on cellulose (File S1). However, the PKA regulatory subunit gene *mcb* (NCU01166) was downregulated in the Δ*cr-1* mutant, and the high-affinity cAMP-PDE gene *acon-2* (NCU00478) was downregulated in the Δ*gna-3* and Δ*cr-1* mutants on cellulose (File S1). It has been shown that Δ*acon-2* mutants have elevated levels of cAMP (45). We previously reported that cAMP-PDE activity is lower in Δ*gna-1* mutants during growth on sucrose, offsetting the reduction in adenylyl cyclase activity in these strains (46). The observed trends in gene expression suggest a compensatory mechanism to increase levels of the free catalytic subunit and PKA activity in the Δ*gna-3* and Δ*cr-1* mutants, which have greatly reduced or undetectable cAMP (47, 48). However, downregulation of *acon-2* and *mcb* cannot overcome the severe defects observed in the absence of the *gna-3* or *cr-1* genes.

### Expression of key cellulase transcriptional regulators is altered in the three mutants

**Fig 5:**
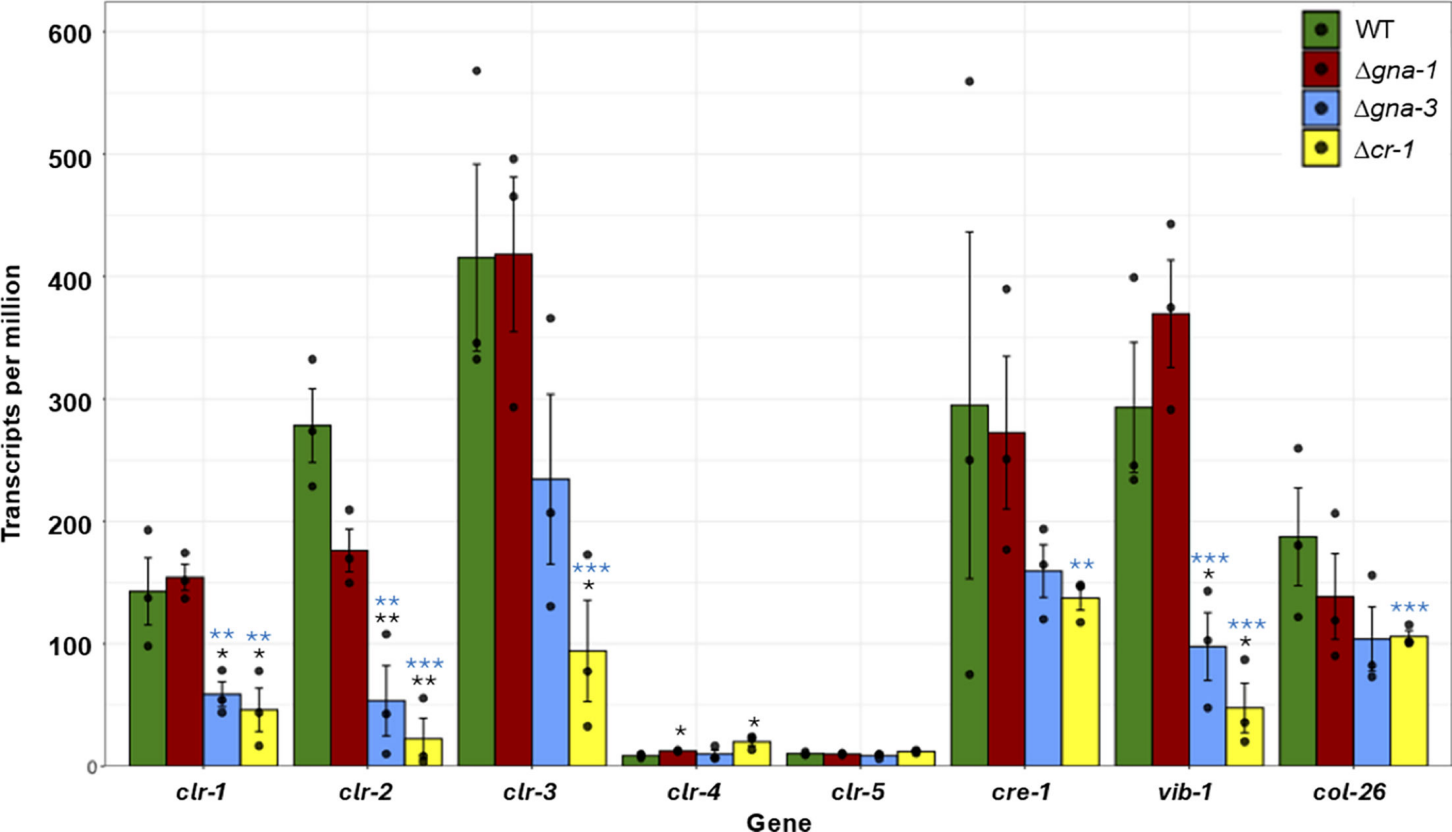
Differential expression of cellulase transcriptional regulators in the three mutants during growth on cellulose. Blue asterisks denote the differentially expressed genes in the mutant vs. wild-type comparison according to the *P*-value from DESeq2. Black asterisks indicate differentially regulated genes determined using a two-tailed Student’s *t*-test. TPMs from three sequencing biological replicates (shown as black dots) were analyzed, and errors are expressed as the standard error. *P < 0.05, **P < 0.01, and ***P < 0.001.

Most of the downregulated genes shared among the mutants in the C-compound and carbohydrate metabolism category belonged to the cellulase enzyme families (Fig. 5). We observed upregulation of the key transcriptional regulators that control the expression of these genes, including *clr-1* (14-fold), *clr-2* (279-fold), *clr-3* (31-fold), *vib-1* (2.7-fold), *col-26* (5.7-fold), and *cre-1* (3.0-fold) in wild type after transfer from glucose to cellulose (File S1). We did not detect differential regulation of *clr-4* or *clr-5* after transfer to cellulose for the wild type in our data set (File S1).

We next investigated possible transcriptional control of these regulatory genes by the Gα subunits and adenylyl cyclase (Fig. 5). Expression of the *cre-1* repressor and *col-26*, encoding a transcription factor that antagonizes *cre-1* expression (18), was uniquely downregulated in Δ*cr-1* mutants. Levels of *vib-1* and *clr-1* mRNAs, encoding transcription factors that act early during the cellulase gene transcriptional pipeline (12, 17), were reduced in Δ*gna-3* and Δ*cr-1* mutants. Transcript levels for *clr-3*, which negatively regulates activation of *clr-1* (13), were lower in Δ*cr-1* mutants relative to wild type. Importantly, *clr-2*, the major transcriptional regulator of cellulase gene expression (12, 17), was downregulated in all three mutants during growth on cellulose; levels in Δ*gna-3* and Δ*cr-1* strains met the twofold DESeq2 cutoff, while those in Δ*gna-1* strains were significantly lower using Student’s *t*-test (Fig. 5).

The results for the Δ*cr-1* strain, which has no detectable cAMP and the greatest reduction in *clr-2* and cellulase gene mRNAs of the three mutants, support the decrease in *cre-1* as a compensatory mechanism. Levels of *clr-4* were significantly elevated in Δ*gna-1* and Δ*cr-1* mutants but did not meet the twofold cutoff (Fig. 5). Since CLR-4 is known to bind the promoter of *cr-1* and to regulate its expression (14), increased levels of *clr-4* could result from a positive feedback loop to induce *cr-1* expression in the Δ*cr-1* mutant. The Δ*gna-1* strain was unique among the three mutants in having reduced expression of *clr-2* but increased levels of *clr-4* (Fig. 5). With regard to the greater amount of *clr-4* mRNA, it is noteworthy that levels of *cr-1* mRNA are significantly elevated in the Δ*gna-1* mutant (*P* value = 0.0003) (File S1).

### Hierarchical clustering reveals nine differentially regulated gene groups in the four strains during growth on cellulose

Most of the above analysis was performed using a twofold cutoff from DEseq on the cellulose expression data. We next focused more closely on a group of 964 genes that were upregulated at least fourfold in wild type during growth on Avicel vs glucose (File S3). Hierarchical clustering was then performed on the log-transformed expression data for these genes in the four strains during growth on cellulose (see Materials and Methods for details). The results revealed nine gene clusters, each with distinct expression patterns (Fig. S2). We focused on several clusters that were most highly expressed in one or two genotypes.

**Fig 6:**
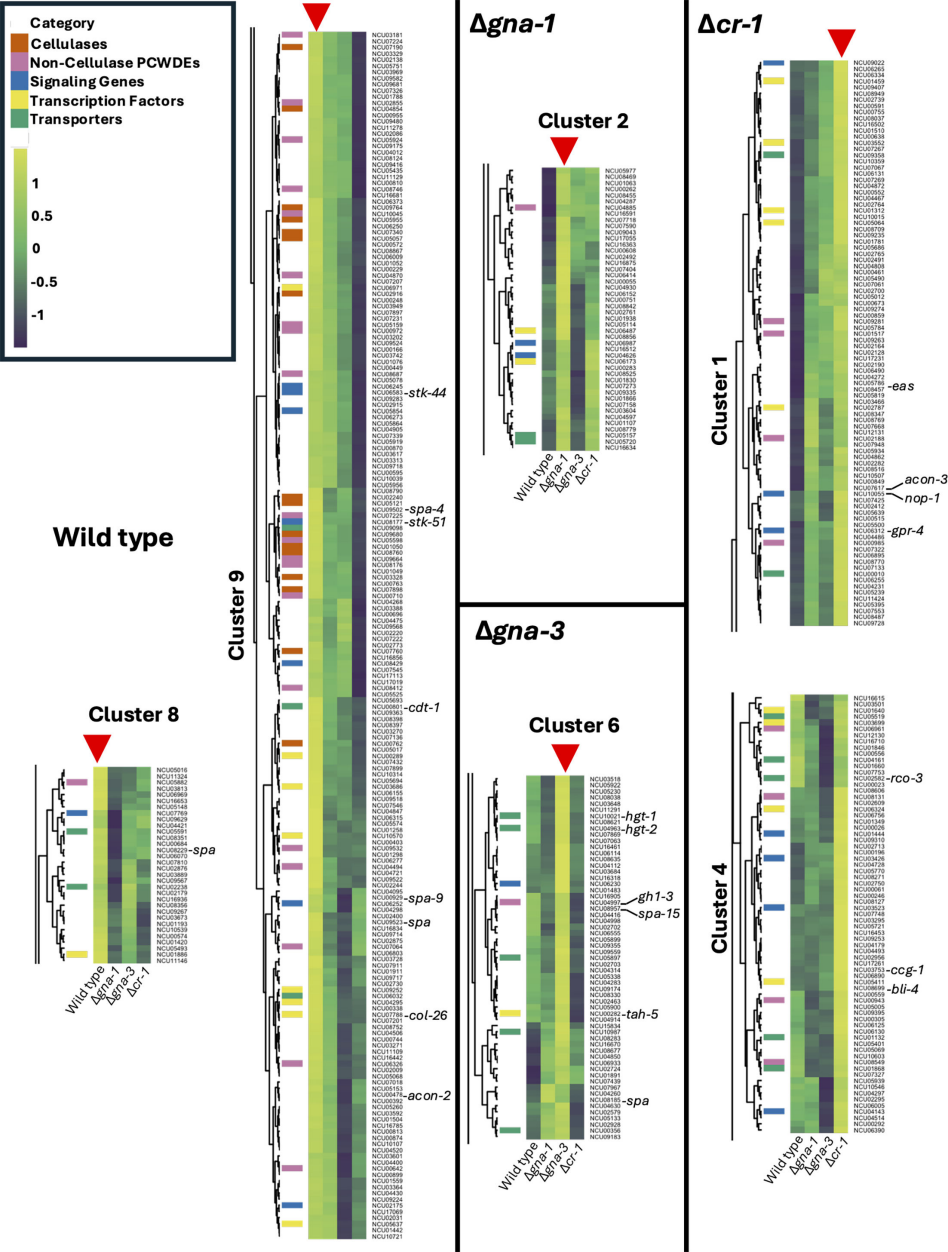
Gene groups identified during hierarchical clustering in wild type and the three mutants during growth on cellulose. A set of 964 genes upregulated at least fourfold in wild type during growth on cellulose vs. glucose was identified. Clustering analysis and heat map generation were performed on the cellulose expression data for these genes from the four strains as described in Materials and Methods. Clusters that were upregulated in ~1 genotype are shown (the complete heatmap with nine clusters is presented in Fig. S1). In each cluster, the genotypes in the lanes from left to right are wild type, Δ*gna-1*, Δ*gna-3,* and Δ*cr-1*. The bars along the left side of the heatmap are color-coded according to the classification of each gene into five major categories, with no shading indicating “Other.” Yellow shading indicates higher relative levels of expression, while green shading reflects lower expression levels. The red arrowhead indicates the lane with the genotype that exhibits the highest expression for that cluster.

Clusters 8 and 9 (230 genes total; Fig. 6) contained genes highly expressed in wild type, including 16 cellulases and 20 non-cellulase PCWDEs that target a variety of substrates. Also present were the high-affinity cAMP-PDE gene *acon-2*, several transcription factors (including *col-26*), four septal pore-associated proteins, two kinases, and the *cdt-1* transporter.

Cluster 2 (47 genes) was enriched in genes that were most highly expressed in the Δ*gna-1* mutant. This relatively small cluster contained two GPCRs, two transcription factors, no cellulases, and only one PCWDE (Fig. 6). Cluster 7 (Fig. S2) was of interest because it contains genes that are expressed more highly in wild type and the Δ*gna-1* mutant relative to the other two strains. This cluster contains most of the cellulase genes that were not included in clusters 8 and 9 (including four β-glucosidases) and the cellulase gene regulators *clr-1*, *clr-2,* and *clr-3*. These observations are consistent with the results discussed above showing that the Δ*gna-1* mutant is less affected in cellulase activity and gene expression than the other two mutants. Cluster 7 also contains 16 PCWDEs, 13 additional transcription factors, and 17 transporters, including the cellobiose transceptor *cdt-2*. Two GPCRs and the VVD photoreceptor were also present in cluster 7.

The 60 genes in cluster 6 were most highly expressed in the Δ*gna-3* mutant (Fig. 6). This cluster contained five transporters, including the high-affinity glucose transporter/transceptors *hgt-1* and *hgt-2*. The elevated expression of these two glucose transport/sensory genes in the Δ*gna-3* mutant may be related to our previous work, suggesting that this strain is “blind” to glucose and could be a compensatory mechanism to bring more glucose into the cell (49). Also in cluster 6 were a serine threonine protein kinase, a transcription factor that regulates aerial hypha height, a xylanase, and two septal pore proteins.

Clusters 1 and 4 (165 genes) were expressed most abundantly in Δ*cr-1*. These clusters contained several non-cellulase PCWDEs and multiple signaling genes, including two serine-threonine protein kinases, one phosphatase, and three GPCRs. The GPCRs included the carbon sensory *gpr-4* and the opsin *nop-1*. Several genes associated with conidiation (including the rodlet gene *eas* and regulatory gene *acon-3*), output from the circadian clock (*ccg-1*), and photobiology (*bli-4* and the opsin GPCR *nop-1*) were also present in these clusters, consistent with the strong conidiation phenotype of the Δ*cr-1* mutant.

### Overexpression of CLR-2 corrects the cellulase defect in the three mutants

**Fig 7:**
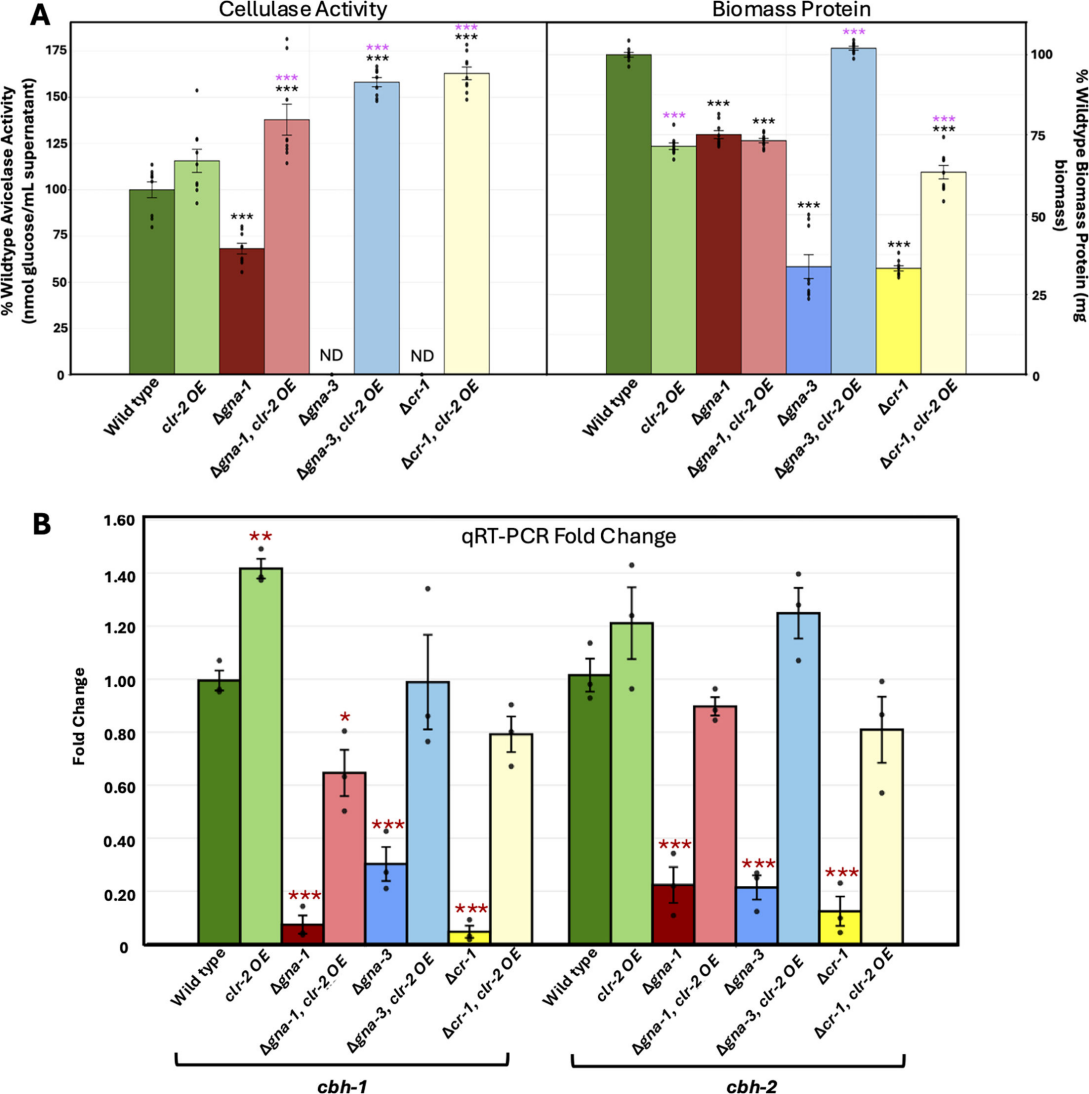
Cellulase activity, biomass protein, and relative expression of two major cellulase genes in wild-type, Δ*gna-1,* Δ*gna-3,* and Δ*cr-1* strains with and without *clr-2* overexpression. (**A**) Cellulase (Avicelase) activity and total biomass protein. A minimum of three biological replicates (black dots) were used for all assays. Statistical significance relative to the wild type without *clr-2* overexpression (clr-2OE) was determined using a two-tailed Student’s *t*-test (black asterisks). The pink asterisks indicate significant differences between strains in the same genetic background with and without *clr-2* overexpression. *P < 0.05; **P < 0.01, and ***P < 0.001. ND, no detectable activity. (**B**) Expression of the cellobiohydrolase genes *cbh-1* and *cbh-2*. Fold change in expression relative to wild type was determined using qRT-PCR data for three biological replicates, each with three technical replicates (average shown by black dots) for wild type, and the three mutants with and without the CLR-2OE construct. Statistical significance relative to the untransformed wild type was determined using a two-tailed Student’s *t*-test (red asterisks). *P* values are indicated as in panel **A**.

As shown above, reduced *clr-2* mRNA levels were common to all three mutants. The Glass laboratory has demonstrated that ectopic overexpression of *clr-2* (*clr-2*OE) using the *ccg-1* promoter leads to cellulase mRNA transcription in wild-type *N. crassa* even when cultured on glucose (19) and that transformation of a cellulase-deficient strain with this construct rescues the cellulase defect (18). To test whether *clr-2* overexpression would rescue cellulase activity in the Δ*gna-1*, Δ*gna-3,* and Δ*cr-1* mutants, we transformed the *clr-2*OE plasmid into the wild-type and mutant strains (Fig. 7A). We found that the cellulase activity in the wild-type *clr-2*OE strain was significantly higher compared to the untransformed wild type, with a 20% increase. In the Δ*gna-1*, *clr-2*OE strain, the cellulase activity was double that of the Δ*gna-1* mutant and 160% of wild type (Fig. 7A). The most dramatic effects were observed in the Δ*gna-3*, *clr-2*OE, and Δ*cr-1*, *clr-2*OE strains, which went from no detectable cellulase activity in the untransformed mutants to activity that was 190% and 163% of the wild type, respectively (Fig. 7A). The Δ*gna-3*, *clr-2*OE, and Δ*cr-1*, *clr-2*OE strains also exhibited large increases in biomass over the corresponding untransformed mutants (Fig. 7A), consistent with the acquisition of significant cellulase activity and more growth on cellulose.

We conducted qRT-PCR to determine whether introduction of the *clr-2*OE construct into our strains led to higher expression of the two most abundantly expressed cellulase genes in wild type on cellulose, *cbh-1*/NCU07340 and *cbh-2*/NCU09680 (Fig. 7B). Relative expression levels of these two genes in the strains without the *clr-2OE* construct were like those obtained from the RNA-seq data, with all three mutants significantly reduced relative to wild type. In contrast, the levels of both *cbh-1* and *cbh-2* were significantly increased in all *clr-2OE* strains relative to the untransformed controls (Fig. 7B). *cbh-1* mRNA was either significantly elevated or similar to wild type in all *clr-2OE* strains except for Δ*gna-1*, *clr-2*OE, which had levels 66.4% of wild type. Amounts of *cbh-2* in the four *clr-2OE* strains were not significantly different from the untransformed wild type (Fig. 7B). Thus, the results from qRT-PCR are consistent with the introduction of the *clr-2OE* construct, leading to increased expression of at least two major cellulase genes in wild type and the three mutants.

## DISCUSSION

This study explored transcriptional regulation by the Gα protein subunits GNA-1, GNA-3, and the adenylyl cyclase CR-1 during growth on cellulose. Previous work from our group investigated the role of heterotrimeric G-proteins in regulating cellulase activity in *N. crassa* (23), with the finding that several G-protein subunits and adenylyl cyclase regulate cellulase activity and the levels of several cellulase mRNAs (23). In this current work, we used genome-wide transcriptional profiling to identify genes that were upregulated in wild type after transfer from glucose to cellulose and that were differentially regulated in the Δ*gna-1*, Δ*gna-3,* and Δ*cr-1* mutants relative to wild type during growth on cellulose.

**Fig 8:**
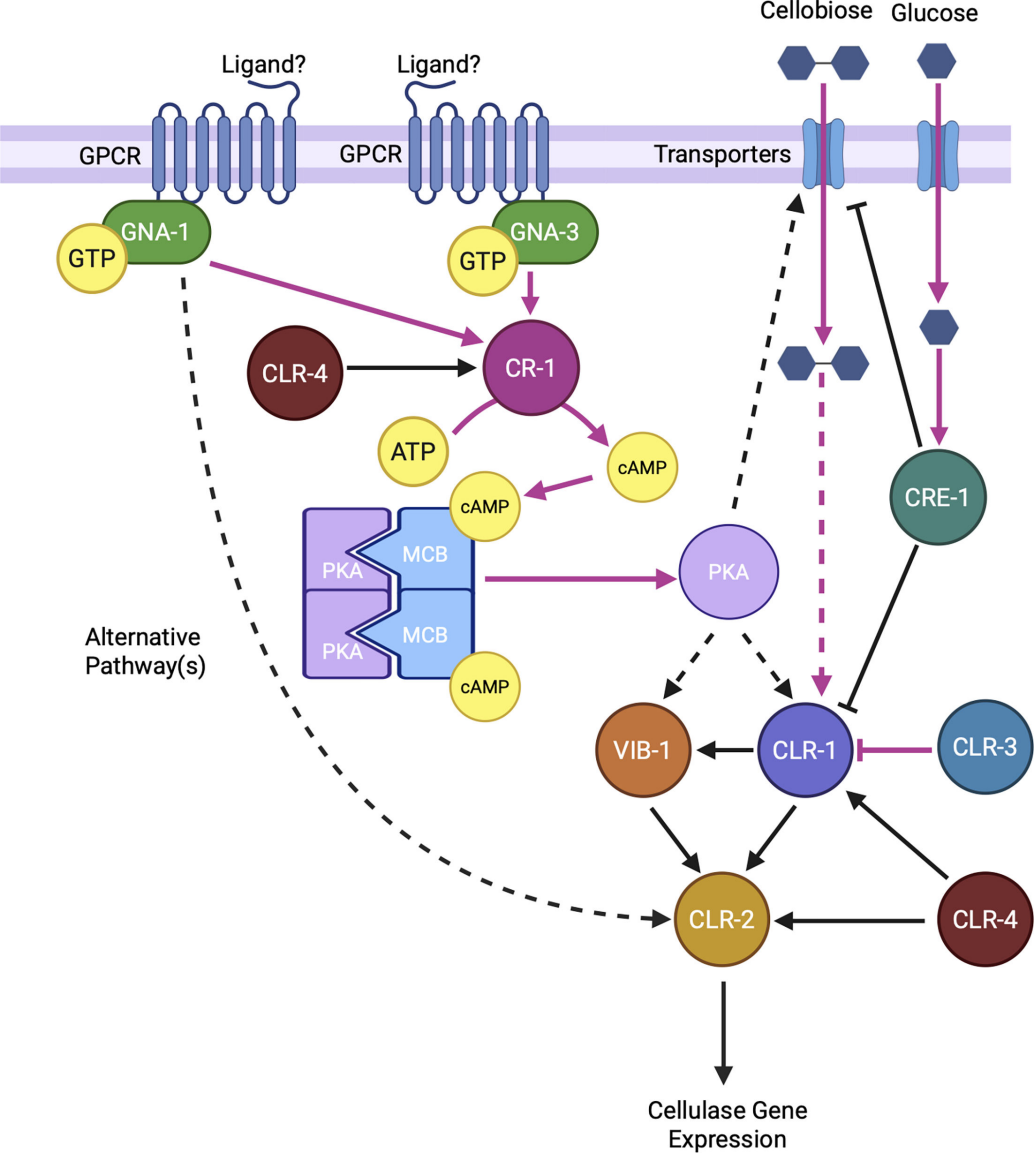
Model for the action of GNA-1, GNA-3, and CR-1/adenylyl cyclase in regulation of cellulase transcription. Black lines indicate transcriptional effects. Magenta lines show post-transcriptional effects, either on protein stability, activity, or binding to small molecules or other proteins. Dotted lines signify indirect linkages. The Gα proteins GNA-1 and GNA-3 increase the activity or abundance (magenta arrows) of adenylyl cyclase (CR-1), resulting in elevated cAMP levels. cAMP binds to the regulatory subunit of PKA (MCB), leading to dissociation of the active catalytic PKA subunit (magenta arrows). This may directly or indirectly (dotted black arrows) lead to expression of one or both transcription factors CLR-1 and VIB-1, which subsequently regulate the expression of the transcription factor CLR-2 (black arrows). CLR-3 is a negative regulator of CLR-1 (magenta arrow), while CLR-4 positively regulates levels of CLR-1, CLR-2, and CR-1 (black arrows). GNA-1-GTP also positively regulates cellulase transcription via an unknown effector pathway(s) that activates CLR-2 (black arrow) or acts post-transcriptionally on CLR-1 or VIB-1. Cellobiose enters the cell using membrane transporters (magenta arrow) and activates CLR-1 via an unknown mechanism (dotted magenta arrow). We propose that cAMP and PKA influence transcription of cellobiose transporters through an unknown mechanism (black dotted arrow). Glucose is also brought into the cell using membrane transporters (magenta arrow) where it activates CRE-1 as a repressor (magenta arrow). CRE-1 inhibits transcription of *clr-1* and cellobiose transporter genes (black arrows) in the presence of glucose. Figure was created using BioRender.

Examination of cellulase transcription factors revealed downregulation of CLR-2 in all three mutants, with the greatest effect in Δ*cr-1*. We obtained evidence that CLR-2 operates downstream of the two Gα proteins and adenylyl cyclase as overexpression of *clr-2* in the mutant backgrounds restored cellulase activity to levels greater than those of the wild type and increased expression of two major cellulase genes (see the model in Fig. 8). The observation that Δ*gna-1* has normal levels of *clr-1* and *vib-1* mRNAs, but reduced *clr-2* mRNA, suggests that GNA-1 acts at a point after *clr-1* transcription to influence *clr-2*. GNA-1 might affect the activity of the CLR-1 protein or some other factor(s) that regulates *clr-2* expression. It is tempting to speculate that GNA-1 is involved in sensing of the cellobiose signal by CLR-1. However, it has been demonstrated that mutational activation of GNA-1 is not sufficient to induce cellulase gene expression in the absence of the inducer cellobiose in *T. reesei* (50), and a similar conclusion was reached for Gna1 in *Chaetomium globosum* (51). Further work is needed to determine how GNA-1 regulates CLR-2 in *N. crassa*.

 We have previously demonstrated that exogenous cAMP rescued cellulase activity and restored several cellulase mRNAs to wild-type levels in Δ*gna-3* and Δ*cr-1* mutants (23). The Δ*cr-1* mutant lacks detectable intracellular cAMP (48). Earlier work in our laboratory showed that GNA-1 stimulates GTP-dependent adenylyl cyclase activity (46) and that levels of cAMP and CR-1 protein are reduced in Δ*gna-3* mutants (47). We also demonstrated that a mutation in the regulatory subunit of PKA (*mcb*) can suppress Δ*gna-1* phenotypes during growth on sucrose (52). Our previous work also supports partial cAMP-independence for GNA-1 in regulation of cellulase gene transcription (23) (Fig. 8). These observations are consistent with a positive role for cAMP in regulating processes downstream of GNA-1, GNA-3, and CR-1, with the most likely candidate being PKA (Fig. 8) (53). Although CLR-1 is phosphorylated on a predicted PKA target sequence, mutation of this site did not influence cellulase activity in *N. crassa* (54, 55). PKA is known to phosphorylate the repressor protein RCM-1/NCU06842, which lessens its binding to the *frq* promoter, thus derepressing *frq* gene expression (56). However, levels of mRNA for *rcm-1* and for the gene encoding its corepressor, *rco-1*, are not misregulated in the mutants. Therefore, the impacts on the expression of cellulase regulators, cellobiose transporters (Fig. 8), and enzymes could result solely from differences in cAMP levels that positively modulate PKA activity and phosphorylation of unknown downstream targets in the mutants in our study.

It is noteworthy that several genes associated with maintenance of CCR in *N. crassa* (18) are downregulated in the Δ*cr-1* mutant (which lacks cAMP), including *cre-1*, *vib-1*, *creB*/NCU08378, and *cre-3*/NCU03887 (File S2). This suggests that cAMP positively impacts CCR in *N. crassa* and that PKA may regulate multiple transcription factors required for the utilization of both glucose and alternative carbon sources, such as cellulose (Fig. 8). The normal expression of the CCR maintenance genes in the Δ*gna-3* mutant, which has cAMP levels ~10% of wild type (47), suggests that the threshold concentration of cAMP that supports CCR gene expression is lower than that for cellulase genes. There are currently no reports that PKA phosphorylates CRE-1 in *N. crassa* (55). However, in *T. reesei*, Cre1 is phosphorylated on Ser241 by casein kinase II (CKII), leading to activation of Cre1 repressor activity (57). We noted that CKII/NCU03124 is upregulated twofold in the Δ*cr-1* mutant (File S2). This would be predicted to increase repression of PCWDE genes and reduce cellulase activity, which is consistent with our results for the Δ*cr-1* mutant.

The upstream regulators of GNA-1 and GNA-3 that control cellulase gene expression are currently unknown. It is tempting to speculate that heterotrimeric Gα proteins may regulate the transport or sensing activity of the cellobiose transceptors CDT-1 and CDT-2 or the glucose transceptors HGT-1, HGT-2, and RCO-3. We previously suggested a possible physical or genetic interaction between GNA-3 and RCO-3, based on the shared asexual sporulation defects in the mutants (47). In addition, as mentioned above, several GPCRs are differentially expressed on cellulose vs glucose and three Pth11 class mutants have phenotypes during growth on cellulose (22). Future studies will investigate both the upstream and downstream signaling components involved in G-protein-mediated regulation of cellulase gene expression in *N. crassa*.

## MATERIALS AND METHODS

### Strains, growth of cultures and cellulase assays

Strains used in this study are indicated in Table S1. Wild-type 74-OR23-1A, Δ*gna-1* 3b10, Δ*gna-3* 31c2, and Δ*cr-1* were used for most analyses. For the growth of cultures, either polycarbonate- or dichlorodimethylsilane (5% vol/vol in chloroform)-treated glass flasks were used to prevent adhesion of hyphae. Macroconidia were inoculated at 1 × 10^6^ cells/mL in 25 mL of Vogel’s minimal medium (VM) (58) with the indicated carbon source at 2% (wt/vol) and then grown in constant light at 25°C with shaking at 200 RPM. Care was taken to only use non-clumping cultures (24).

For isolation of cell-free supernatants, cultures were grown in VM with 2% glucose for 16 h, washed with VM with no carbon, and then transferred to VM with 2% Avicel for 2 days. Isolation of cell-free supernatants and extraction of total cellular protein from the cell pads was performed as described (23). For isolation of total RNA, cultures were grown and washed as described for cell-free supernatants, except that the cultures were grown in VM with 2% glucose for 12 h and transferred to VM with 2% glucose or 2% Avicel for 4 h. Cultures were collected, and total RNA was isolated as described in reference 59.

Cellulase (Avicelase) and protein assays were performed as previously described (23).

### Transcriptomics

Total RNA was used to prepare libraries using Illumina standard protocols and sequenced using a NextSeq 2000, HiSeq 4000, or NovaSeq 6000, with at least 20 M reads generated for each sample (Illumina, San Diego, CA, USA). Sequencing data from three biological replicates for each strain cultured on a carbon source were analyzed. Raw reads were mapped against an index file generated from the current assembly of the *N. crassa* genome via the Broad Institute using Kallisto version 0.46.1 (60) to generate raw counts and TPMs. PCA was performed using RStudio (https://wwwR-project.org/) to check the agreement between the biological replicates for each strain. Differential expression analysis was performed on raw counts using DESeq2 version 1.26.0 (61) (https://github.com/thelovelab/DESeq2/blob/devel/NEWS). Genes with at least a twofold change and *P*-values less than 0.05 were considered differentially expressed. Differentially expressed genes were then filtered to set expression as 0 in strains with fewer than 10 TPMs for all three replicates. FungiFun2 (https://fungifun3.hki-jena.de/) was used to perform Functional Catalog (FunCat) analysis of the regulated genes in each genotype relative to wild type.

A group of 964 genes that were upregulated at least fourfold in wild type during growth on cellulose vs glucose was compiled from the RNA-seq data. TPMs for these genes in the four strains grown on cellulose were log-transformed to normalize variance. Hierarchical clustering analysis was then performed using Euclidean distance as the similarity metric and the complete linkage method for cluster generation using pheatmap (V1.0.13) (62) in R (63).

### Overexpression of the *clr-2* transcription factor

Strains with the *mus-51::bar*^R^ and *his-3* mutations in the wild-type background were available in our laboratory (Table S1), while those carrying the *mus-51::bar*^R^ and *his-3* mutations in the Δ*gna-1,* Δ*gna-3*, and Δ*cr-1* mutant backgrounds were constructed using sexual crosses (64) (Table S1). Vector Pc*2* (19), which expresses *clr-2* under the control of the *ccg-1* promoter from the *his-3* locus (*clr-2*OE), was a gift from N. L. Glass. The recipient strains were electroporated with vector Pc2, as previously described (65), with selection on the medium lacking histidine. Transformants were purified to homokaryons by streak-plating on the medium lacking histidine three times. DNA from the purified transformants was checked using diagnostic PCRs, and those that contained the p*ccg-1::clr-2* insert at the *his-3* locus and did not amplify a band corresponding to the intact *his-3* locus were tested for cellulase activity, as described above. Quantitative reverse transcriptase PCR was performed on RNA isolated from the wild-type and *clr-2*OE strains using methods and primers described in reference 23, except that NCU02364 was used as the control gene instead of actin. In contrast to actin, the expression of NCU02364 is similar in wild type and the three mutants (File S1).

## SUPPLEMENTAL MATERIAL

File S1 mbio.03720-25-s0001.xlsx TPMs and DESeq results.

File S2 mbio.03720-25-s0002.xlsx DESeq for certain gene groups.

File S3 mbio.03720-25-s0003.xlsx Results of hierarchical clustering analysis of log-transformed TPMs.

Supplemental Material mbio.03720-25-s0004.pdf Supplemental tables and figures.

ASM does not own the copyrights to Supplemental Material that may be linked to, or accessed through, an article. The authors have granted ASM a non-exclusive, world-wide license to publish the Supplemental Material files. Please contact the corresponding author directly for reuse.
