# Supplementary material for: Heterotrimeric G-proteins and cAMP regulate gene expression during growth on cellulose in Neurospora crassa
Source: mBio. 2026 Jan 15;17(2):e03720-25. doi: 10.1128/mbio.03720-25 (PMC12892961; doi:10.1128/mbio.03720-25)
Supplement: Supplemental Material — Supplemental tables and figures. [file mbio.03720-25-s0004.pdf]

Supplementary Material for Collier et al.

Table S1. Strains used in this study.

Strain Name	Genotype	Comments	Source/ Reference
74-OR23-1A	Wild type, *mat A*	Wild type	FGSC[a]
OR8-1a	Wild type, *mat a*	Wild type	FGSC[a]
3b10	Δ*gna-1*::*hph, mat a*	Δ*gna-1* (NCU06493)	(45)
31c2	Δ*gna-3*::*hph, mat A*	Δ*gna-3* (NCU05206)	(46)
Δ*cr-1*	Δ*cr-1*::*hph, mat a*	Δ*cr-1* (NCU08377)	(22)
FGSC9717	Δ*mus-51::bar, his-3, mat A*	*his-3* recipient for *clr-2*OE vector	FGSC[a]
KAB3522, #7	Δ*gna-1*::*hph,* Δ*mus-51::bar,, his-3, mat a*	Δ*gna-1, his-3* recipient for *clr-2*OE vector	This study
KAB3523, 4-5	Δ*gna-3*::*hph,* Δ*mus-51::bar, his-3, mat a*	Δ*gna-3, his-3* recipient for *clr-2*OE vector	This study
KAB3524, 2.1D	Δ*cr-1*::*hph,* Δ*mus-51::bar,, his-3, mat a*	Δ*cr-1, his-3* recipient for *clr-2*OE vector	This study
KAB3536, R3P1	Δ*mus-52::bar,* p*ccg-1*::*clr-2*::*his-3*$^+$*, mat A*	Wild type with *clr-2*OE	This study
KAB3516, 1341-1	Δ*gna-1*::*hph,* Δ*mus-51::bar,* p*ccg-1*::*clr-2*::*his-3*$^+$*, mat a*	Δ*gna-1* with *clr-2*OE	This study
KAB3518, 3431-3	Δ*gna-3*::*hph,* Δ*mus-51::bar,* p*ccg-1*::*clr-2*::*his-3*$^+$*, mat a*	Δ*gna-3* with *clr-2*OE	This study
KAB3538, 2.4	Δ*cr-1*::*hph,* Δ*mus-51::bar,* p*ccg-1*::*clr-2*::*his-3*$^+$*, mat a*	Δ*cr-1* with *clr-2*OE	This study

[a] Fungal Genetics Stock Center, Kansas State University, Manhattan, KS.

Table S2. Expression of predicted GPCRs and photoreceptors in mutants relative to wild type during growth on cellulose

NCU	Gene Name	Δ*gna-1*	Δ*gna-3*	Δ*cr-1*
**G-Protein Coupled Receptors**				
**NCU Number**	**Gene Name**	**Δ*gna-1***	**Δ*gna-3***	**Δ*cr-1***
NCU10055	*nop-1*	2.37	ND	6.94
NCU01735	*orp-1*	-4.67	19.49	ND
NCU00138	*pre-1*	NE	NE	NE
NCU05758	*pre-2*	NE	NE	ND
NCU00786	*gpr-1*	NE	NE	NE
NCU04626	*gpr-2*	ND	NE	2.01
NCU09427	*gpr-3*	ND	ND	-3.90
NCU06312	*gpr-4*	ND	NE	6.46
NCU00300	*gpr-5*	ND	ND	ND
NCU09195	*gpr-6*	ND	2.17	2.08
NCU09883	*gpr-7*	ND	ND	ND
NCU03253	*gpr-8*	-2.14	ND	-2.40
NCU03238	*gpr-9*	ND	-2.04	-2.64
NCU04987	*gpr-10*	ND	ND	ND
NCU00182	*gpr-11*	ND	ND	ND
NCU00005	*gpr-12*	ND	ND	ND
NCU06629	*gpr-13*	NE	NE	NE
NCU06987	*gpr-14*	ND	ND	ND
NCU00700	*gpr-15*	NE	NE	NE
NCU02903	*gpr-16*	NE	NE	NE
NCU04106	*gpr-17*	ND	ND	ND
NCU04931	*gpr-18*	2.24	ND	ND
NCU05101	*gpr-19*	NE	NE	NE
NCU05187	*gpr-20*	NE	NE	NE

NCU05189	*gpr-21*	13.74	NE	3.54
NCU05307	*gpr-22*	ND	ND	2.36
NCU05829	*gpr-23*	-3.54	ND	-5.24
NCU05854	*gpr-24*	ND	ND	-3.28
NCU06531	*gpr-25*	NE	NE	NE
NCU17171	*gpr-26*	NE	NE	NE
NCU07538	*gpr-27*	NE	NE	NE
NCU16721	*gpr-28*	NE	NE	NE
NCU07649	*gpr-29*	ND	ND	ND
NCU07769	*gpr-30*	ND	ND	ND
NCU08429	*gpr-31*	ND	ND	-3.25
NCU08431	*gpr-32*	NE	NE	NE
NCU08447	*gpr-33*	ND	ND	ND
NCU08624	*gpr-34*	ND	-7.57	-16.36
NCU08718	*gpr-35*	NE	NE	NE
NCU09022	*gpr-36*	ND	ND	2.76
NCU09201	*gpr-37*	4.41	ND	3.60
NCU09796	*gpr-38*	NE	NE	NE
NCU09823	*gpr-39*	ND	2.23	4.69
**Photoreceptors**				
NCU02356	*wc-1*	ND	2.31	ND
NCU10055	*nop-1*	2.37	ND	6.9
NCU03967	*vvd*	ND	ND	ND
NCU00582	*cry*	ND	3.98	6.6
NCU04834	*phy-1*	ND	ND	3
NCU05790	*phy-2*	ND	ND	ND

ND = Not Differentially Expressed; NE= Not Expressed

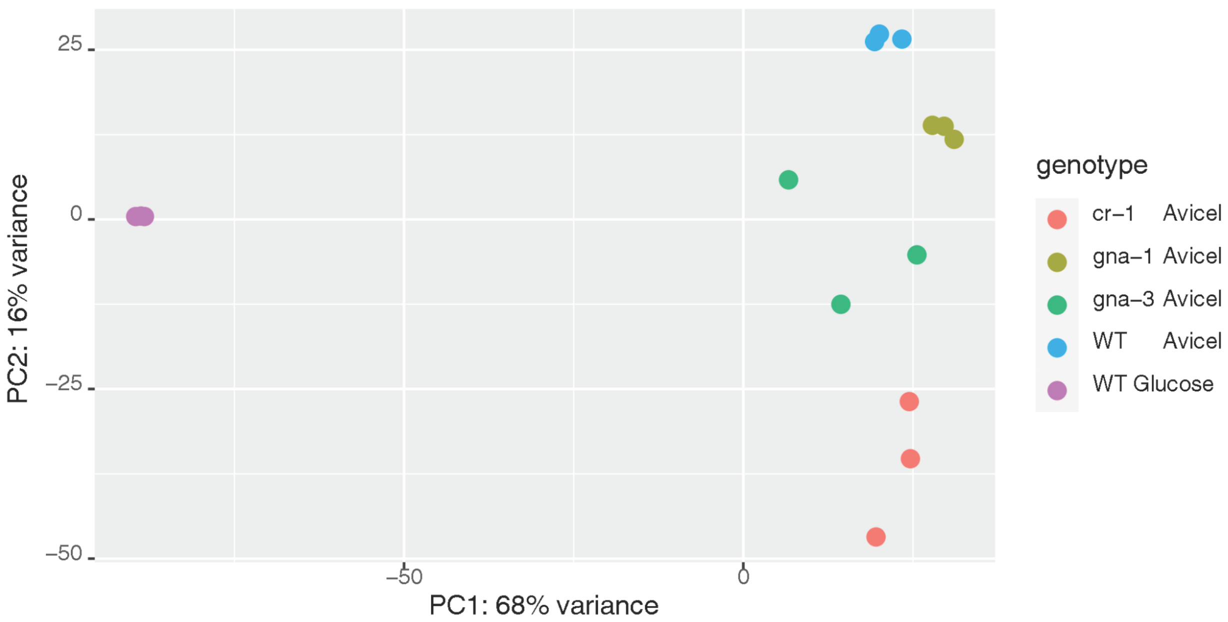

Figure S1. Principal component analysis of RNAseq data. Principal Component Analysis (PCA) was performed in R (https://wwwR-project.org/) utilizing data from the Kallisto 0.46.1 alignment of RNAseq reads to the reference genome *Neurospora crassa* OR74A. Data included reads for three biological replicates for each of the four strains on the indicated carbon sources. WT= Wild type, cr-1= Δ*cr-1*, gna-1= Δ*gna-1* and gna-3= Δ*gna-3*.

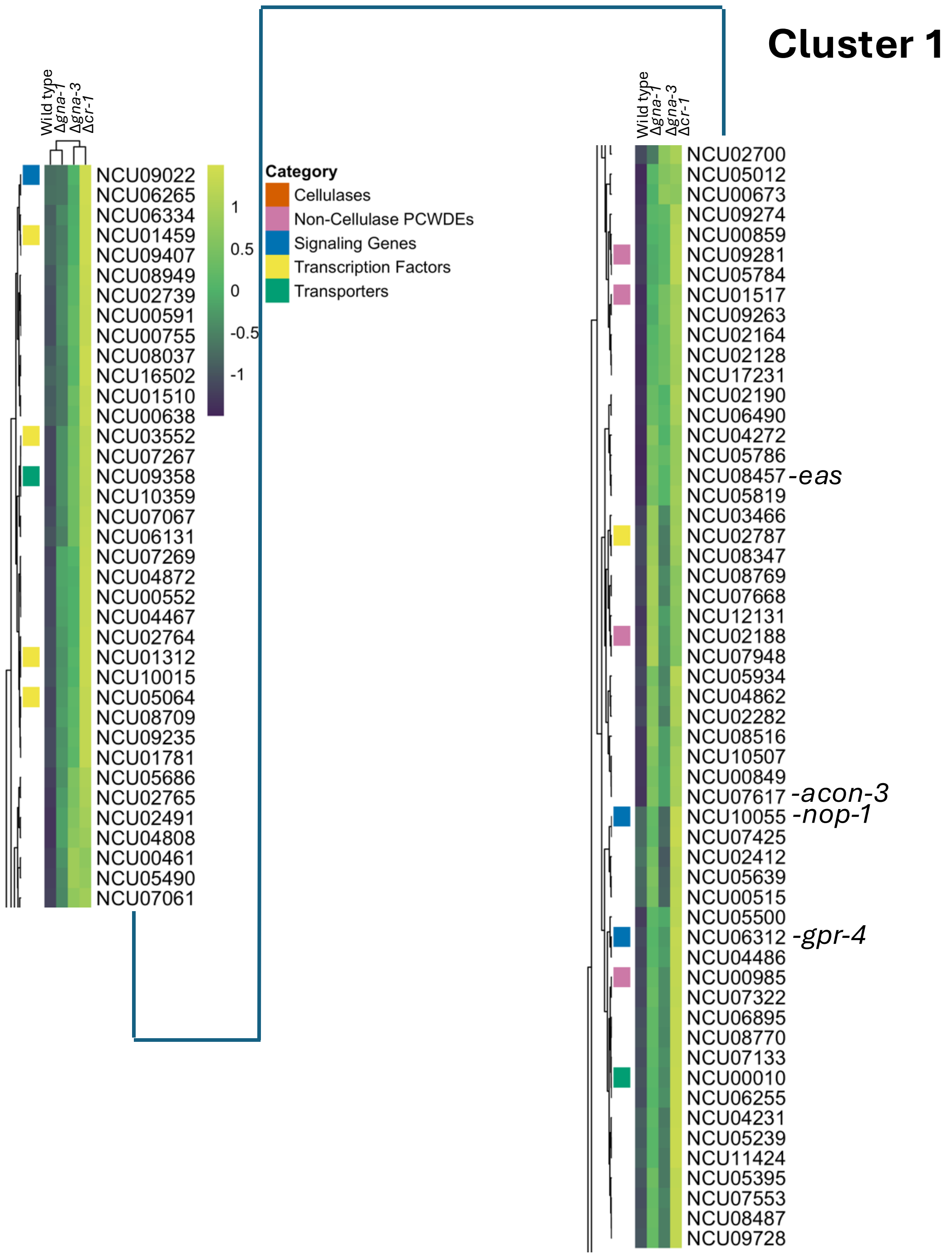
Cluster 1
Wild type
Δgna-1
Δgna-3
Δcr-1
Category
Cellulases
Non-Cellulase PCWDEs
Signaling Genes
Transcription Factors
Transporters
1
0.5
0
-0.5
-1
NCU09022
NCU06265
NCU06334
NCU01459
NCU09407
NCU08949
NCU02739
NCU00591
NCU00755
NCU08037
NCU16502
NCU01510
NCU00638
NCU03552
NCU07267
NCU09358
NCU10359
NCU07067
NCU06131
NCU07269
NCU04872
NCU00552
NCU04467
NCU02764
NCU01312
NCU10015
NCU05064
NCU08709
NCU09235
NCU01781
NCU05686
NCU02765
NCU02491
NCU04808
NCU00461
NCU05490
NCU07061
NCU02700
NCU05012
NCU00673
NCU09274
NCU00859
NCU09281
NCU05784
NCU01517
NCU09263
NCU02164
NCU02128
NCU17231
NCU02190
NCU06490
NCU04272
NCU05786
NCU08457 -eas
NCU05819
NCU03466
NCU02787
NCU08347
NCU08769
NCU07668
NCU12131
NCU02188
NCU07948
NCU05934
NCU04862
NCU02282
NCU08516
NCU10507
NCU00849
NCU07617 -acon-3
NCU10055 -nop-1
NCU07425
NCU02412
NCU05639
NCU00515
NCU05500
NCU06312 -gpr-4
NCU04486
NCU00985
NCU07322
NCU06895
NCU08770
NCU07133
NCU00010
NCU06255
NCU04231
NCU05239
NCU11424
NCU05395
NCU07553
NCU08487
NCU09728

Cluster 2

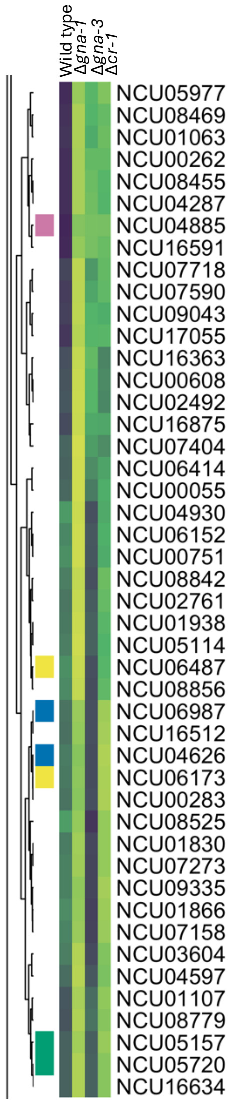

Wild type
Δgna-1
Δgna-3
Δcr-1
NCU05977
NCU08469
NCU01063
NCU00262
NCU08455
NCU04287
NCU04885
NCU16591
NCU07718
NCU07590
NCU09043
NCU17055
NCU16363
NCU00608
NCU02492
NCU16875
NCU07404
NCU06414
NCU00055
NCU04930
NCU06152
NCU00751
NCU08842
NCU02761
NCU01938
NCU05114
NCU06487
NCU08856
NCU06987
NCU16512
NCU04626
NCU06173
NCU00283
NCU08525
NCU01830
NCU07273
NCU09335
NCU01866
NCU07158
NCU03604
NCU04597
NCU01107
NCU08779
NCU05157
NCU05720
NCU16634

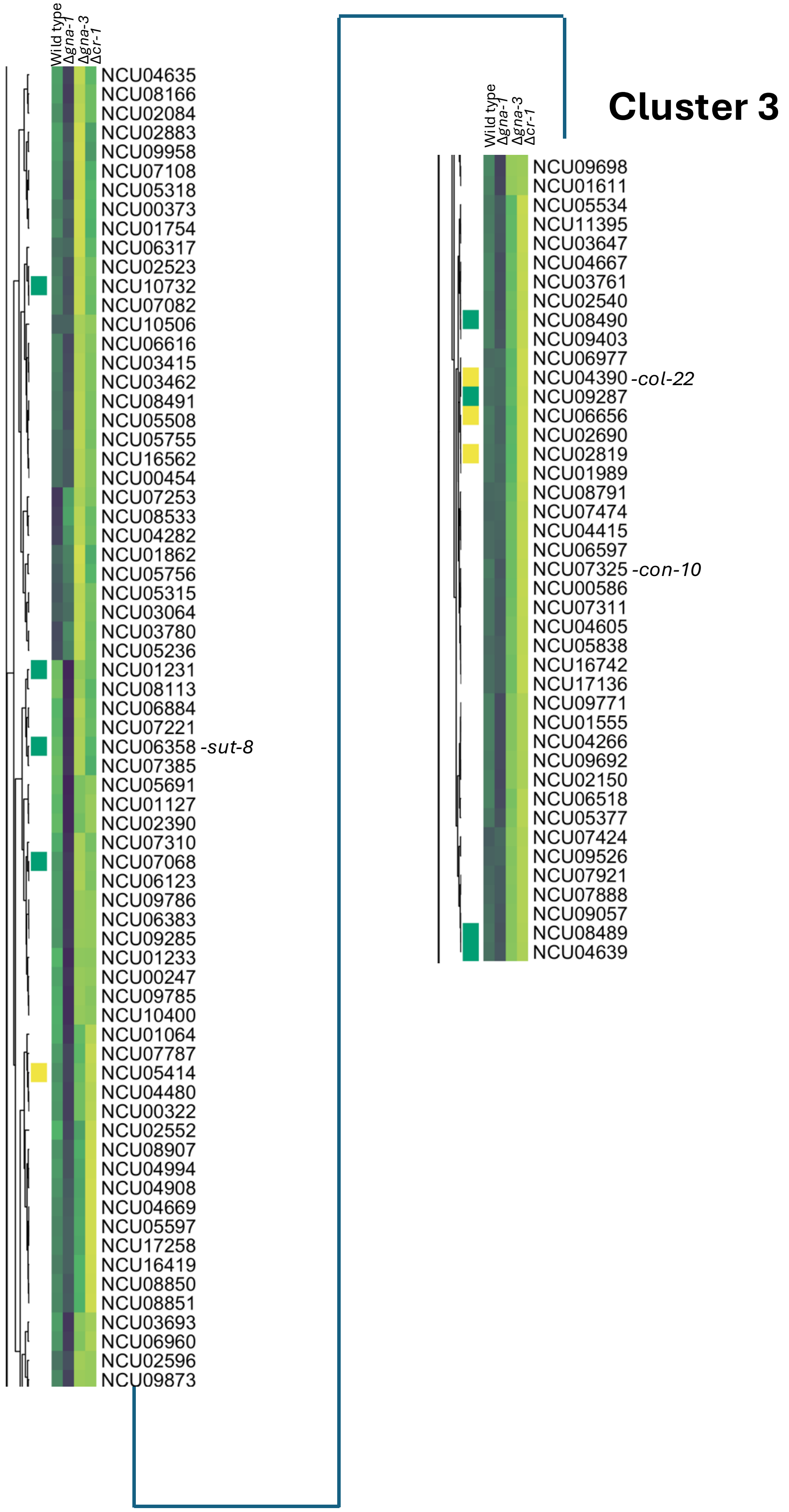

Cluster 3
Wild type
Δgna-1
Δgna-3
Δcr-1
NCU04635
NCU08166
NCU02084
NCU02883
NCU09958
NCU07108
NCU05318
NCU00373
NCU01754
NCU06317
NCU02523
NCU10732
NCU07082
NCU10506
NCU06616
NCU03415
NCU03462
NCU08491
NCU05508
NCU05755
NCU16562
NCU00454
NCU07253
NCU08533
NCU04282
NCU01862
NCU05756
NCU05315
NCU03064
NCU03780
NCU05236
NCU01231
NCU08113
NCU06884
NCU07221
NCU06358 -sut-8
NCU07385
NCU05691
NCU01127
NCU02390
NCU07310
NCU07068
NCU06123
NCU09786
NCU06383
NCU09285
NCU01233
NCU00247
NCU09785
NCU10400
NCU01064
NCU07787
NCU05414
NCU04480
NCU00322
NCU02552
NCU08907
NCU04994
NCU04908
NCU04669
NCU05597
NCU17258
NCU16419
NCU08850
NCU08851
NCU03693
NCU06960
NCU02596
NCU09873
Wild type
Δgna-1
Δgna-3
Δcr-1
NCU09698
NCU01611
NCU05534
NCU11395
NCU03647
NCU04667
NCU03761
NCU02540
NCU08490
NCU09403
NCU06977
NCU04390 -col-22
NCU09287
NCU06656
NCU02690
NCU02819
NCU01989
NCU08791
NCU07474
NCU04415
NCU06597
NCU07325 -con-10
NCU00586
NCU07311
NCU04605
NCU05838
NCU16742
NCU17136
NCU09771
NCU01555
NCU04266
NCU09692
NCU02150
NCU06518
NCU05377
NCU07424
NCU09526
NCU07921
NCU07888
NCU09057
NCU08489
NCU04639

Cluster 4

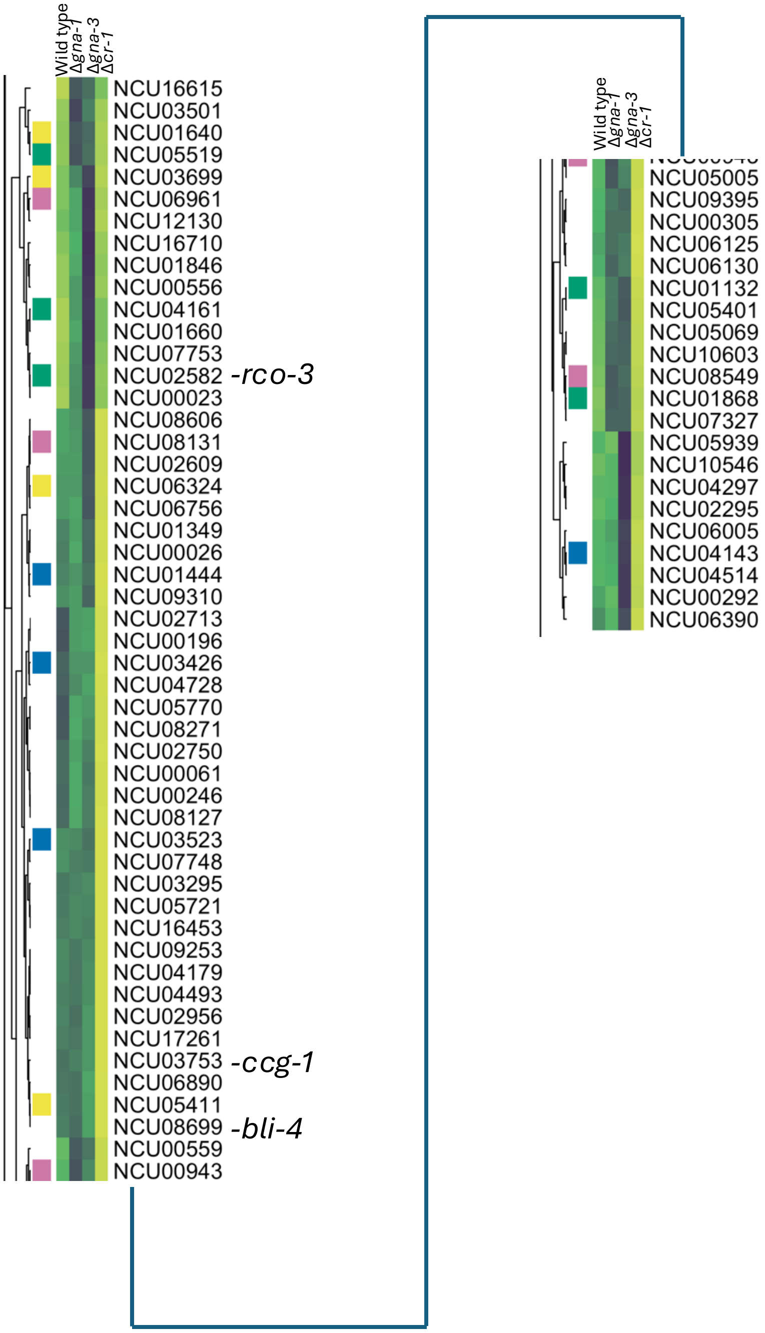
Wild type
Δgna-1
Δgna-3
Δcr-1
NCU16615
NCU03501
NCU01640
NCU05519
NCU03699
NCU06961
NCU12130
NCU16710
NCU01846
NCU00556
NCU04161
NCU01660
NCU07753
NCU02582 -rco-3
NCU00023
NCU08606
NCU08131
NCU02609
NCU06324
NCU06756
NCU01349
NCU00026
NCU01444
NCU09310
NCU02713
NCU00196
NCU03426
NCU04728
NCU05770
NCU08271
NCU02750
NCU00061
NCU00246
NCU08127
NCU03523
NCU07748
NCU03295
NCU05721
NCU16453
NCU09253
NCU04179
NCU04493
NCU02956
NCU17261
NCU03753 -ccg-1
NCU06890
NCU05411
NCU08699 -bli-4
NCU00559
NCU00943
Wild type
Δgna-1
Δgna-3
Δcr-1
NCU05005
NCU09395
NCU00305
NCU06125
NCU06130
NCU01132
NCU05401
NCU05069
NCU10603
NCU08549
NCU01868
NCU07327
NCU05939
NCU10546
NCU04297
NCU02295
NCU06005
NCU04143
NCU04514
NCU00292
NCU06390

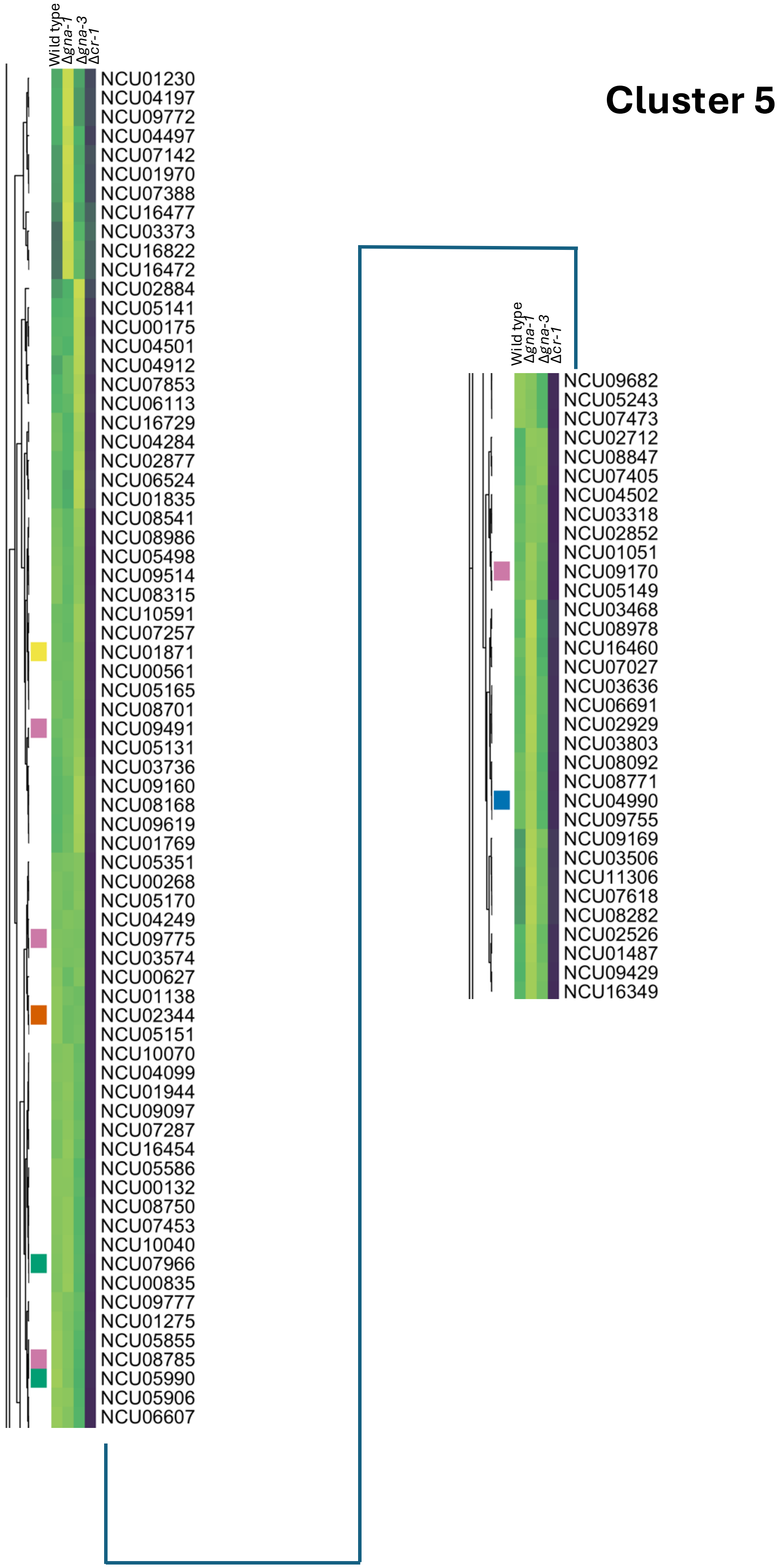

Cluster 5
Wild type
Δgna-1
Δgna-3
Δcr-1
NCU01230
NCU04197
NCU09772
NCU04497
NCU07142
NCU01970
NCU07388
NCU16477
NCU03373
NCU16822
NCU16472
NCU02884
NCU05141
NCU00175
NCU04501
NCU04912
NCU07853
NCU06113
NCU16729
NCU04284
NCU02877
NCU06524
NCU01835
NCU08541
NCU08986
NCU05498
NCU09514
NCU08315
NCU10591
NCU07257
NCU01871
NCU00561
NCU05165
NCU08701
NCU09491
NCU05131
NCU03736
NCU09160
NCU08168
NCU09619
NCU01769
NCU05351
NCU00268
NCU05170
NCU04249
NCU09775
NCU03574
NCU00627
NCU01138
NCU02344
NCU05151
NCU10070
NCU04099
NCU01944
NCU09097
NCU07287
NCU16454
NCU05586
NCU00132
NCU08750
NCU07453
NCU10040
NCU07966
NCU00835
NCU09777
NCU01275
NCU05855
NCU08785
NCU05990
NCU05906
NCU06607
Wild type
Δgna-1
Δgna-3
Δcr-1
NCU09682
NCU05243
NCU07473
NCU02712
NCU08847
NCU07405
NCU04502
NCU03318
NCU02852
NCU01051
NCU09170
NCU05149
NCU03468
NCU08978
NCU16460
NCU07027
NCU03636
NCU06691
NCU02929
NCU03803
NCU08092
NCU08771
NCU04990
NCU09755
NCU09169
NCU03506
NCU11306
NCU07618
NCU08282
NCU02526
NCU01487
NCU09429
NCU16349

Cluster 6

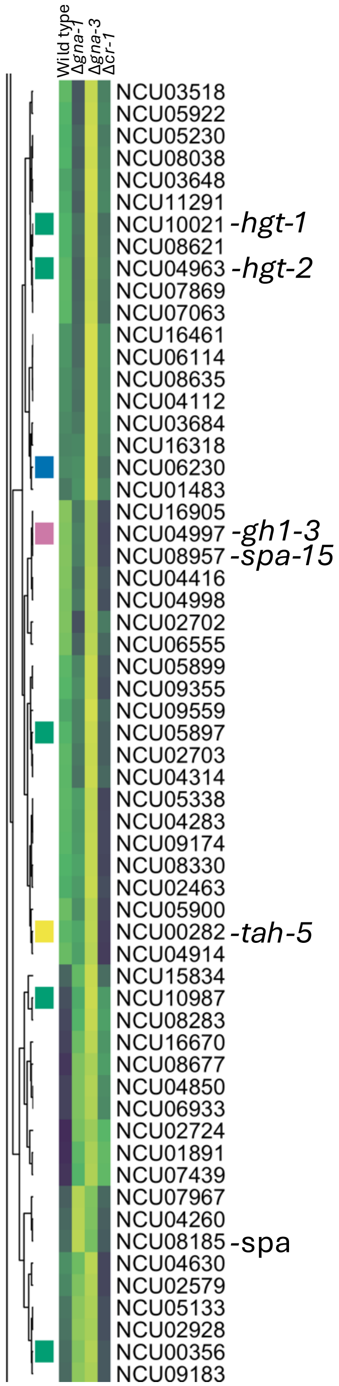

Wild type
Δgna-1
Δgna-3
Δcr-1
NCU03518
NCU05922
NCU05230
NCU08038
NCU03648
NCU11291
NCU10021 -hgt-1
NCU08621
NCU04963 -hgt-2
NCU07869
NCU07063
NCU16461
NCU06114
NCU08635
NCU04112
NCU03684
NCU16318
NCU06230
NCU01483
NCU16905
NCU04997 -gh1-3
NCU08957 -spa-15
NCU04416
NCU04998
NCU02702
NCU06555
NCU05899
NCU09355
NCU09559
NCU05897
NCU02703
NCU04314
NCU05338
NCU04283
NCU09174
NCU08330
NCU02463
NCU05900
NCU00282 -tah-5
NCU04914
NCU15834
NCU10987
NCU08283
NCU16670
NCU08677
NCU04850
NCU06933
NCU02724
NCU01891
NCU07439
NCU07967
NCU04260
NCU08185 -spa
NCU04630
NCU02579
NCU05133
NCU02928
NCU00356
NCU09183

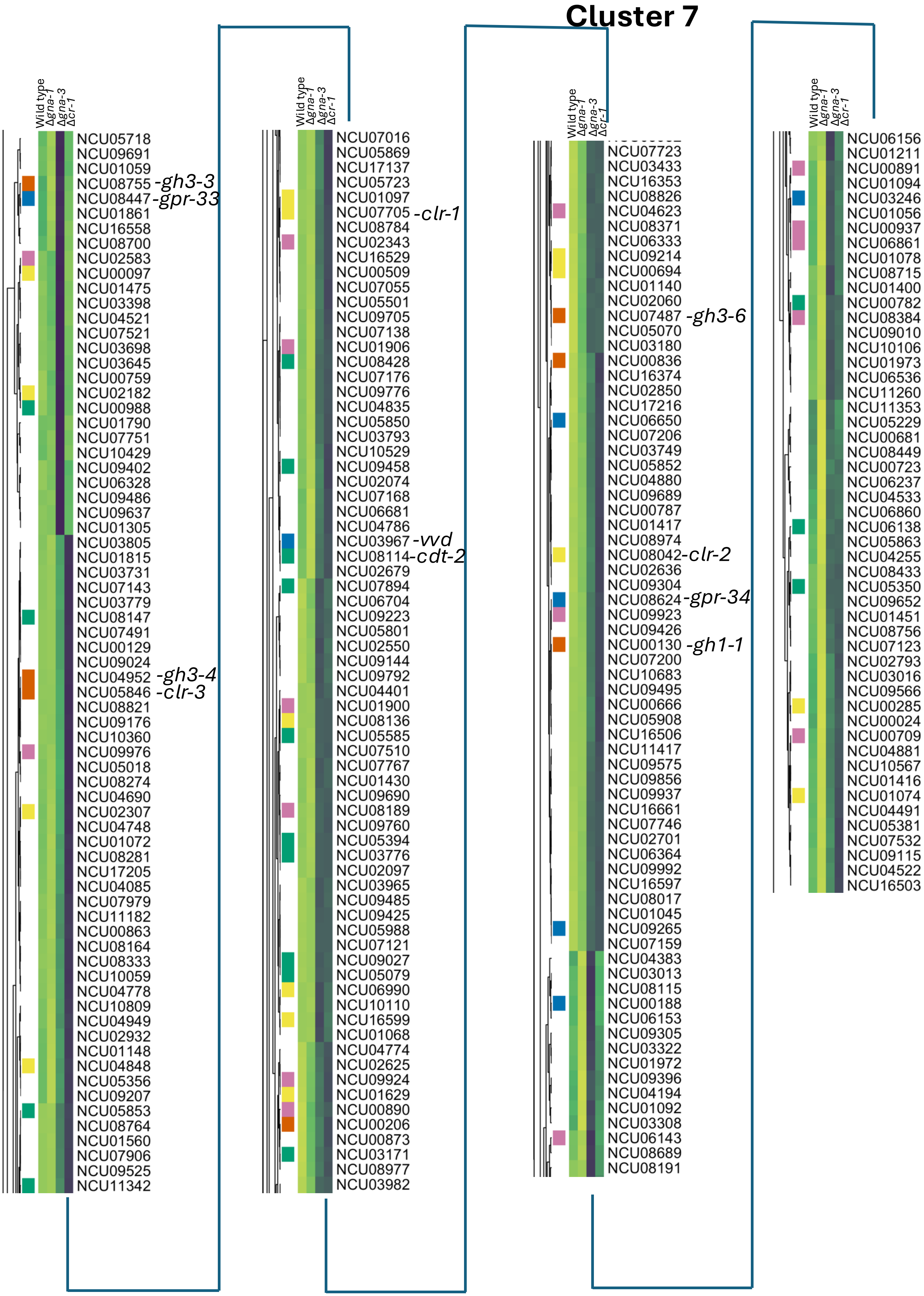
Cluster 7
Wild type
Δgna-1
Δgna-3
Δcr-1
NCU05718
NCU09691
NCU01059
NCU08755 -gh3-3
NCU08447-gpr-33
NCU01861
NCU16558
NCU08700
NCU02583
NCU00097
NCU01475
NCU03398
NCU04521
NCU07521
NCU03698
NCU03645
NCU00759
NCU02182
NCU00988
NCU01790
NCU07751
NCU10429
NCU09402
NCU06328
NCU09486
NCU09637
NCU01305
NCU03805
NCU01815
NCU03731
NCU07143
NCU03779
NCU08147
NCU07491
NCU00129
NCU09024
NCU04952 -gh3-4
NCU05846 -clr-3
NCU08821
NCU09176
NCU10360
NCU09976
NCU05018
NCU08274
NCU04690
NCU02307
NCU04748
NCU01072
NCU08281
NCU17205
NCU04085
NCU07979
NCU11182
NCU00863
NCU08164
NCU08333
NCU10059
NCU04778
NCU10809
NCU04949
NCU02932
NCU01148
NCU04848
NCU05356
NCU09207
NCU05853
NCU08764
NCU01560
NCU07906
NCU09525
NCU11342
Wild type
Δgna-1
Δgna-3
Δcr-1
NCU07016
NCU05869
NCU17137
NCU05723
NCU01097
NCU07705 -clr-1
NCU08784
NCU02343
NCU16529
NCU00509
NCU07055
NCU05501
NCU09705
NCU07138
NCU01906
NCU08428
NCU07176
NCU09776
NCU04835
NCU05850
NCU03793
NCU10529
NCU09458
NCU02074
NCU07168
NCU06681
NCU04786
NCU03967 -vvd
NCU08114-cdt-2
NCU02679
NCU07894
NCU06704
NCU09223
NCU05801
NCU02550
NCU09144
NCU09792
NCU04401
NCU01900
NCU08136
NCU05585
NCU07510
NCU07767
NCU01430
NCU09690
NCU08189
NCU09760
NCU05394
NCU03776
NCU02097
NCU03965
NCU09485
NCU09425
NCU05988
NCU07121
NCU09027
NCU05079
NCU06990
NCU10110
NCU16599
NCU01068
NCU04774
NCU02625
NCU09924
NCU01629
NCU00890
NCU00206
NCU00873
NCU03171
NCU08977
NCU03982
Wild type
Δgna-1
Δgna-3
Δcr-1
NCU07723
NCU03433
NCU16353
NCU08826
NCU04623
NCU08371
NCU06333
NCU09214
NCU00694
NCU01140
NCU02060
NCU07487 -gh3-6
NCU05070
NCU03180
NCU00836
NCU16374
NCU02850
NCU17216
NCU06650
NCU07206
NCU03749
NCU05852
NCU04880
NCU09689
NCU00787
NCU01417
NCU08974
NCU08042-clr-2
NCU02636
NCU09304
NCU08624-gpr-34
NCU09923
NCU09426
NCU00130 -gh1-1
NCU07200
NCU10683
NCU09495
NCU00666
NCU05908
NCU16506
NCU11417
NCU09575
NCU09856
NCU09937
NCU16661
NCU07746
NCU02701
NCU06364
NCU09992
NCU16597
NCU08017
NCU01045
NCU09265
NCU07159
NCU04383
NCU03013
NCU08115
NCU00188
NCU06153
NCU09305
NCU03322
NCU01972
NCU09396
NCU04194
NCU01092
NCU03308
NCU06143
NCU08689
NCU08191
Wild type
Δgna-1
Δgna-3
Δcr-1
NCU06156
NCU01211
NCU00891
NCU01094
NCU03246
NCU01056
NCU00937
NCU06861
NCU01078
NCU08715
NCU01400
NCU00782
NCU08384
NCU09010
NCU10106
NCU01973
NCU06536
NCU11260
NCU11353
NCU05229
NCU00681
NCU08449
NCU00723
NCU06237
NCU04533
NCU06860
NCU06138
NCU05863
NCU04255
NCU08433
NCU05350
NCU09652
NCU01451
NCU08756
NCU07123
NCU02793
NCU03016
NCU09566
NCU00285
NCU00024
NCU00709
NCU04881
NCU10567
NCU01416
NCU01074
NCU04491
NCU05381
NCU07532
NCU09115
NCU04522
NCU16503

Cluster 8

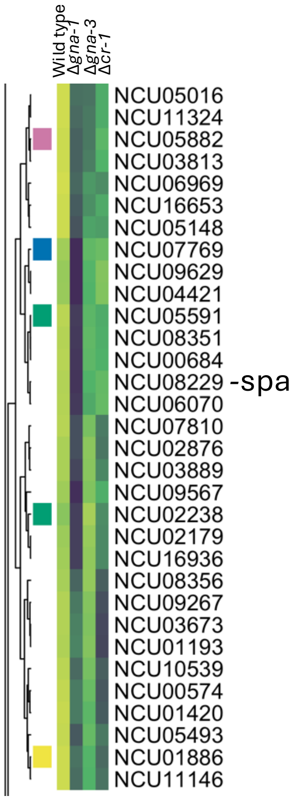
Wild type
Δgna-1
Δgna-3
Δcr-1
NCU05016
NCU11324
NCU05882
NCU03813
NCU06969
NCU16653
NCU05148
NCU07769
NCU09629
NCU04421
NCU05591
NCU08351
NCU00684
NCU08229 -spa
NCU06070
NCU07810
NCU02876
NCU03889
NCU09567
NCU02238
NCU02179
NCU16936
NCU08356
NCU09267
NCU03673
NCU01193
NCU10539
NCU00574
NCU01420
NCU05493
NCU01886
NCU11146

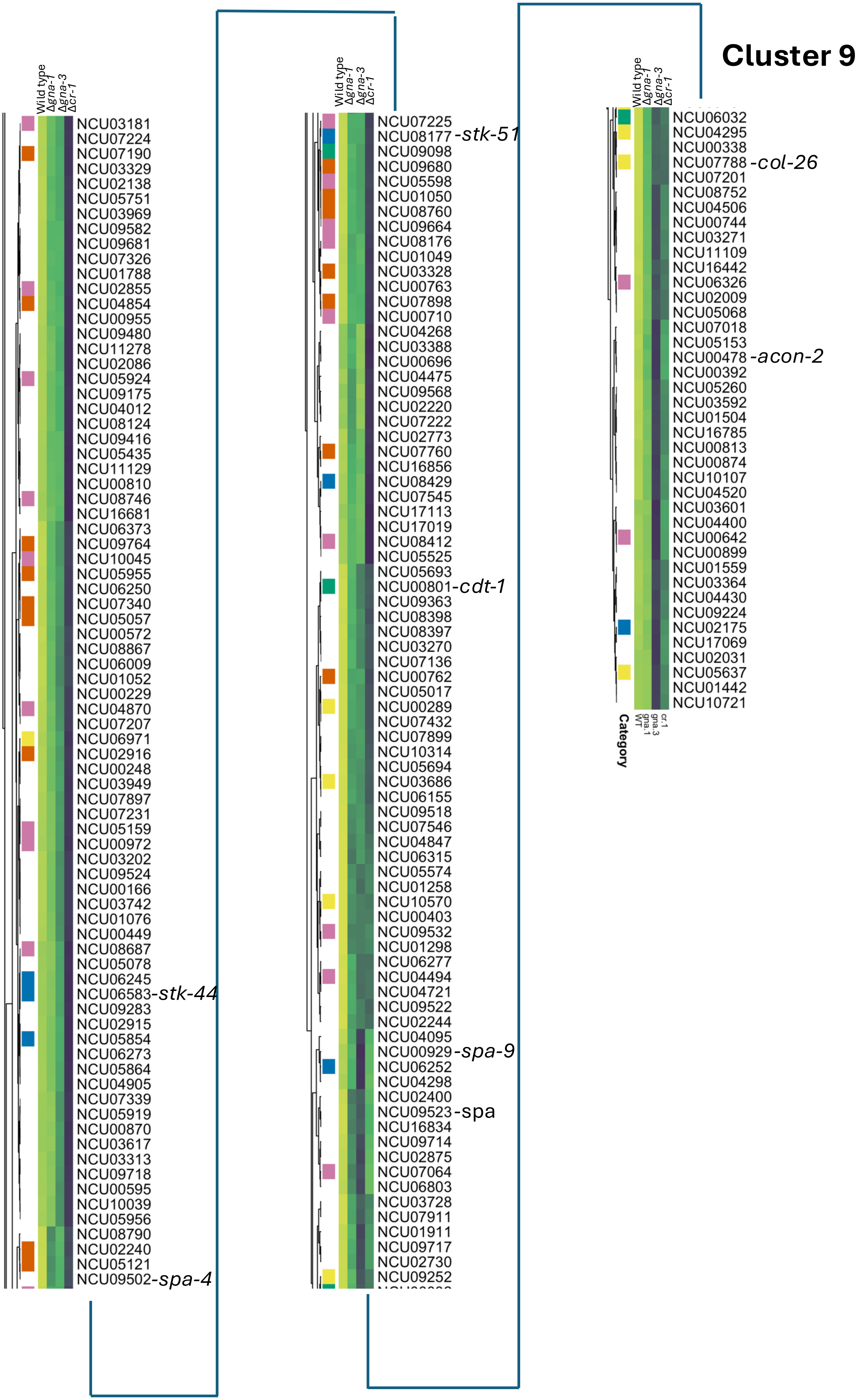
Cluster 9
Wild type
Δgna-1
Δgna-3
Δcr-1
NCU03181
NCU07224
NCU07190
NCU03329
NCU02138
NCU05751
NCU03969
NCU09582
NCU09681
NCU07326
NCU01788
NCU02855
NCU04854
NCU00955
NCU09480
NCU11278
NCU02086
NCU05924
NCU09175
NCU04012
NCU08124
NCU09416
NCU05435
NCU11129
NCU00810
NCU08746
NCU16681
NCU06373
NCU09764
NCU10045
NCU05955
NCU06250
NCU07340
NCU05057
NCU00572
NCU08867
NCU06009
NCU01052
NCU00229
NCU04870
NCU07207
NCU06971
NCU02916
NCU00248
NCU03949
NCU07897
NCU07231
NCU05159
NCU00972
NCU03202
NCU09524
NCU00166
NCU03742
NCU01076
NCU00449
NCU08687
NCU05078
NCU06245
NCU06583-stk-44
NCU09283
NCU02915
NCU05854
NCU06273
NCU05864
NCU04905
NCU07339
NCU05919
NCU00870
NCU03617
NCU03313
NCU09718
NCU00595
NCU10039
NCU05956
NCU08790
NCU02240
NCU05121
NCU09502-spa-4
Wild type
Δgna-1
Δgna-3
Δcr-1
NCU07225
NCU08177-stk-51
NCU09098
NCU09680
NCU05598
NCU01050
NCU08760
NCU09664
NCU08176
NCU01049
NCU03328
NCU00763
NCU07898
NCU00710
NCU04268
NCU03388
NCU00696
NCU04475
NCU09568
NCU02220
NCU07222
NCU02773
NCU07760
NCU16856
NCU08429
NCU07545
NCU17113
NCU17019
NCU08412
NCU05525
NCU05693
NCU00801-cdt-1
NCU09363
NCU08398
NCU08397
NCU03270
NCU07136
NCU00762
NCU05017
NCU00289
NCU07432
NCU07899
NCU10314
NCU05694
NCU03686
NCU06155
NCU09518
NCU07546
NCU04847
NCU06315
NCU05574
NCU01258
NCU10570
NCU00403
NCU09532
NCU01298
NCU06277
NCU04494
NCU04721
NCU09522
NCU02244
NCU04095
NCU00929-spa-9
NCU06252
NCU04298
NCU02400
NCU09523-spa
NCU16834
NCU09714
NCU02875
NCU07064
NCU06803
NCU03728
NCU07911
NCU01911
NCU09717
NCU02730
NCU09252
Wild type
Δgna-1
Δgna-3
Δcr-1
NCU06032
NCU04295
NCU00338
NCU07788-col-26
NCU07201
NCU08752
NCU04506
NCU00744
NCU03271
NCU11109
NCU16442
NCU06326
NCU02009
NCU05068
NCU07018
NCU05153
NCU00478-acon-2
NCU00392
NCU05260
NCU03592
NCU01504
NCU16785
NCU00813
NCU00874
NCU10107
NCU04520
NCU03601
NCU04400
NCU00642
NCU00899
NCU01559
NCU03364
NCU04430
NCU09224
NCU02175
NCU17069
NCU02031
NCU05637
NCU01442
NCU10721
Category
WT
gna.1
gna.3
cr.1

Figure S2. Clusters from heatmap resulting from hierarchical clustering of genes expressed in wild type and the three mutants during growth on cellulose. A set of 964 genes was identified that was upregulated at least four-fold in wild type during growth on cellulose vs. glucose. Clustering analysis and heat map generation were performed on the log-transformed cellulose expression data for these genes from the four strains as described in the Materials and Methods. In each cluster, genotypes from left to right are wild type, Δ*gna-1*, Δ*gna-3* and Δ*cr-1*. Yellow shading indicates higher relative levels of expression, while green shading reflects lower expression levels. The bars along the left side of the heatmap are color-coded according to classification of each gene into five major categories, with no shading indicating "Other." Each page of the figure contains genes in one of the clusters, with green lines connecting the parts of large clusters that would not contiguously fit on one page.
